# Understanding the Relationship between Pressure and Temperature Unfolding of Proteins

**DOI:** 10.1021/jacsau.5c00185

**Published:** 2025-03-20

**Authors:** Christian Roumestand, Erika Dudas, Rita Puglisi, Antonino Calió, Philippe Barthe, Piero Andrea Temussi, Annalisa Pastore

**Affiliations:** † Centre de Biologie Structurale, CNRS UMR 5048, INSERM U1054, Université de Montpellier, 34090 Montpellier, France; ‡ European Synchrotron Radiation Facility, Ave des Martyrs, 38000 Grenoble, France; § King’s College London, 5 Cutcombe Rd, SE59RT London, U.K.; ∥ University Grenoble Alps, 38000 Grenoble, France; ⊥ Department of Chemistry, Universita’ Federico II, 80100 Napoli, Italy

**Keywords:** frataxin, high-pressure nuclear magnetic resonance, thermodynamic stability, protein folding, Yfh1

## Abstract

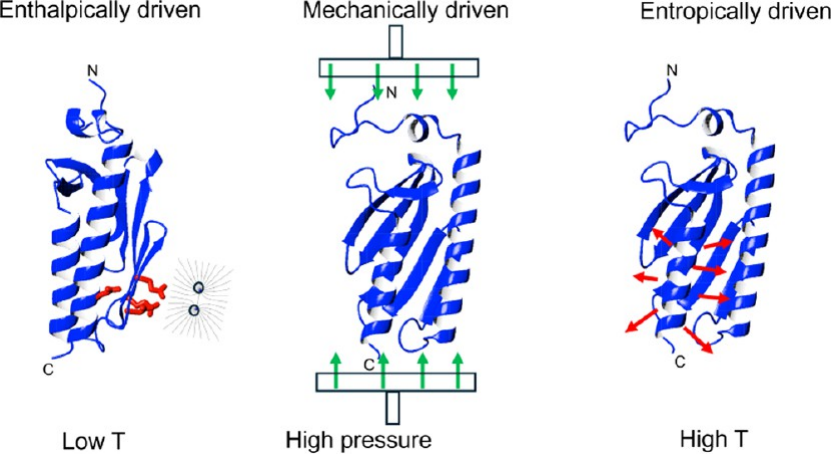

Proteins unfold under different environmental insults, among which are heat, cold, high pressure, and chaotropic agents. Understanding the mechanisms that determine unfolding under each of these conditions is an important problem that directly relates to the physical forces that determine the three-dimensional structure of a protein. Here, we studied a residue-specific description of the unfolding transitions of marginally stable yeast protein Yfh1 using high-pressure nuclear magnetic resonance. We compared the cold, heat, and pressure unfolded states and demonstrated what has up to now been only a hypothesis: the pressure-unfolded spectrum at room temperature shares features in common with that at low but not at high temperature and room pressure, suggesting a tighter similarity of the mechanisms and a similar role of hydration in these two processes. By exploring the phase diagram of the protein and mapping unfolding onto the three-dimensional structure of the protein, we also show that the pressure-induced unfolding pathways at low and high temperatures differ, suggesting a synergic mechanism between pressure- and temperature-induced denaturation. Our observations help us to reconstruct the structural events determining unfolding and distinguish the mechanisms that rule the different processes of unfolding.

## Introduction

The study of protein unfolding, which corresponds to the process of loss of tertiary structure acquired after the amino acid chain has come out from the ribosome, has fascinated and keeps fascinating generations of scientists because its elucidation holds the promise of understanding the forces that determine protein structure formation. However, structure may be lost, both in vivo and in vitro, in several different ways, so that speaking about a unique unfolded state is utterly meaningless (refs (1 and 2) and references within). In nature, unfolding can be triggered by loss/gain of post-translational modifications, mutations, or any imbalance in the environmental conditions (e.g., changes in pH, protein concentration, crowding, confinement, etc.). In vitro, unfolding can be triggered by changes in the temperature, pH, solvent composition, and/or pressure. Among these causes, pressure-induced denaturation is probably the process relatively less studied, possibly because of the nontrivial experimental problems posed by pressurization. The process remains nevertheless extremely interesting both because some proteins do experience unusually high pressure in nature (e.g., proteins from organisms that live in the deep seas) and because the transition may involve atomic forces probably less dominant in other unfolding processes but not for this less important one. Additionally, high pressure presents the advantage to move cold denaturation, an important unfolding transition in principle experienced by all proteins but most often hindered in practice by water freezing,^3^ to observable temperatures.^4^ Together, these considerations call for a more attentive and detailed study of the mechanisms involved in pressure-induced unfolding.

Despite the pioneering work of several researchers (refs (5–15) just to cite a few), several aspects of pressure-induced unfolding remain unclear. Most studies of protein unfolding caused by pressure show, for instance, that it is necessary to reach high values of pressure (of the order of thousands of bars), often coadjuvated by mild concentrations of caotropic solvents, before unfolding can be observed for proteins from common piezo-tolerant or pressure mesophile organisms. This is at variance with the devastating effects that comparatively less drastic temperature increases may induce in proteins from common thermally mesophile organisms. It is thus important to understand pressure unfolding on a protein that does not need the addition of cosolvents and kbars to unfold.

We have recently studied the pressure unfolding of a marginally stable protein, Yfh1 from *Saccharomyces cerevisiae*, that unfolds at pressures so low that its unfolding does not present the typical lag phase usually observed before a protein loses its fold.^16^ Yfh1 is a small protein (123 residues in its mature, full-length form) that is highly conserved from bacteria to primates and interesting under several aspects: when depleted of salt, it is possible to observe its cold denaturation at temperatures above water freezing in solutions under quasi-physiological conditions (278 K or 5 °C) and without the need of introducing any destabilizing mutation or chaotropic agent.^17^ The high-temperature unfolding is also relatively low (around 308 K or 35 °C). At room temperature, Yfh1 is in equilibrium between folded and unfolded forms, with the population of the unfolded form ∼30%. This is clearly visible in the NMR spectra, in which two sets of resonances are observed. Addition of even small quantities of salts causes disappearance of the unfolded form,^18^ indicating that the two forms are in a slow equilibrium in the NMR time scale. The Yfh1 fold consists of two N- and C-terminal helices that pack against a 5–7 strand β-sheet depending on the orthologue.^19^

**Figure 1:**
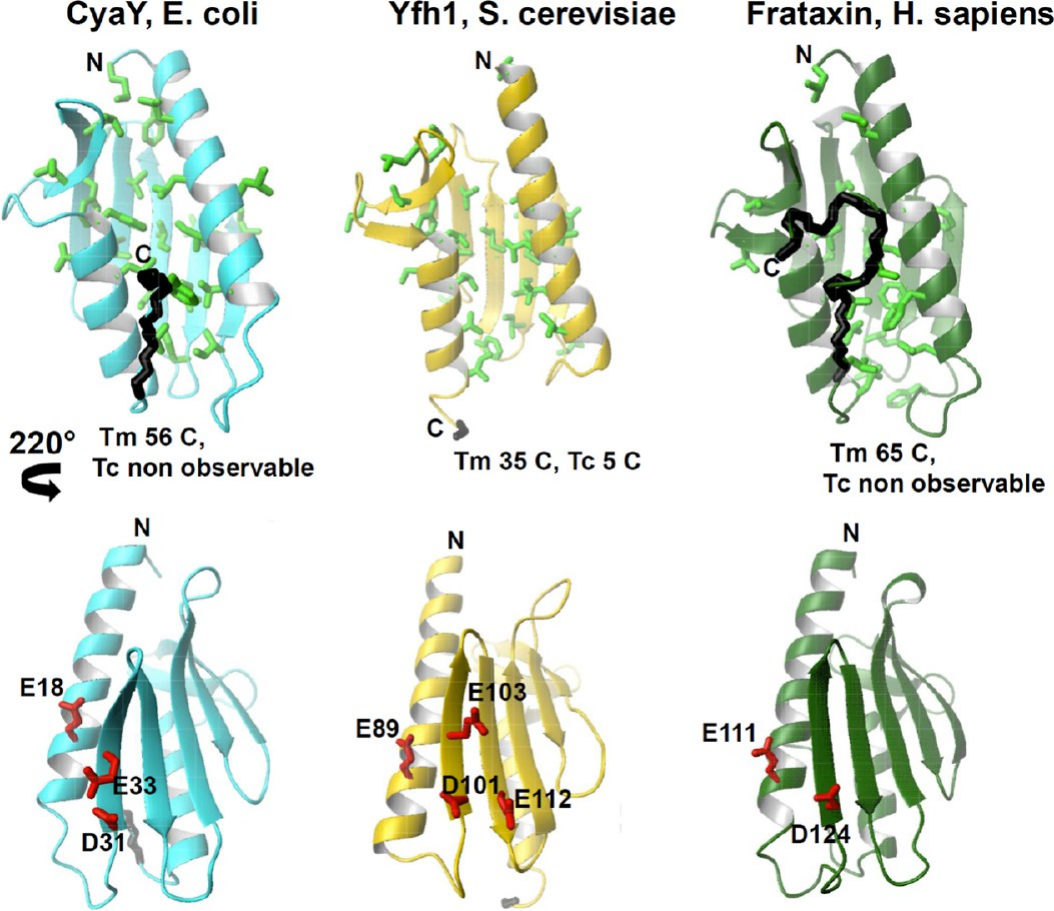
Comparison between the structures of Yfh1 and its bacterial and human orthologues highlighting features that correlate with their stabilities. Top: the structures of these proteins consist in two helices packed again a β-sheet. When long enough, the C-terminus inserts in between the two helices and protects the hydrophobic core. Yfh1 has a C-terminus much shorter than the other two orthologues leaving the core more exposed. We have previously demonstrated that shortening of the bacterial protein by three residues leads to a loss of 14 °C, while lengthening of Yfh1 by four residues leads to a gain of 8 °C on the high-temperature transition midpoint (Adinolfi et al., 2004^20^). Bottom: the same structures rotated by 220° around the *y* axis, showing a cluster of negatively charged residues (in red). These residues create electrostatic repulsion that have been demonstrated to determine observation of cold denaturation at detectable temperatures: mutation of even one of them shifts the low-temperature transition while negligibly affecting the high-temperature transition. The PDB codes used were 1ew4, 2ga5, and 1egk for *Escherichia coli*, *S. cerevisiae*, and *Homo sapiens* frataxins, respectively. Notice that the structure of Yfh1 has a disordered N-terminal region, which was omitted here for clarity.

We have found two features in the past that greatly affect the stability of Yfh1. First, the length of the C-terminus is shorter as compared to other orthologues and the melting temperatures correlate with the length of this secondary structure element^20^ (Figure 1, top). This is because when present, the C-terminus inserts between the two terminal helices protecting the hydrophobic core. We have demonstrated that shortening other orthologues causes a major destabilization, whereas lengthening the C-terminus of Yfh1 leads to an increase of the melting temperature.^20^ Second, Yfh1, but not other orthologues, contains four negative residues in the first helix and second strands^21^ (Figure 1, bottom). This quadrilateral of negative charges causes significant electrostatic repulsion, which leads to cold denaturation at observable temperatures under conditions in which hydrophobic forces are weaker: mutation of only one of these residues to a neutral hydrophilic group leads to the shift of cold denaturation at temperatures below the water freezing point while not significantly affecting the high-temperature transition.^21^ Having a system for which we understand in detail the elements that determine protein stability makes Yfh1 a precious tool that we have extensively exploited to probe the mechanisms that determine protein unfolding.^22^

In a previous study, we determined the phase diagram of Yfh1 unfolding as a function of pressure (1–5000 bar) and temperature 278–313 K (5–40 °C), both in the absence and in the presence of fold stabilizers using the intrinsic fluorescence of the two tryptophan residues of Yfh1.^16^ We demonstrated that Yfh1 has a much higher sensitivity to pressure than most of the proteins previously used for high pressure unfolding studies: 50% unfolding occurs already at pressures around 100 bar at room temperature without any cosolvent. For comparison, other globular proteins, such as the immunoglobulin-like module of titin I27 or a hyper-stable variant of Staphylococcus nuclease, need 2–3 kbar and mild concentrations of guanidinium chloride to unfold.^23,24^ The arginine binding protein from *Thermotoga maritima* (ArgBP) undergoes minor structural changes at 10 kbar.^25^

In this study, we exploited these unusual properties to study the pressure-unfolding pathways of Yfh1 as a function of temperature by nuclear magnetic resonance (NMR). This technique is almost unique in providing residue-specific information on the folding/unfolding pathways of proteins.^26,27^ We compared the properties of the high pressure-unfolded state with the unfolded states at low and high temperatures and room atmosphere. From our observations, we can draw direct experimental conclusions on the role of hydration on the unfolded state at high pressure, demonstrating a closer resemblance of the cold and pressure-induced unfolded states as compared to the high-temperature one. This conclusion resonates with several past experimental and theoretical important publications.^28−32^ We also unexpectedly found that, at mild pressures, the temperature-induced unfolding processes are less cooperative, indicating a synergic mechanism between temperature and pressure. Under these conditions, we can directly identify the unfolding pathways at low and high temperatures. This possibility allowed us to show that the two pathways are different and reflect our previous hypotheses on the forces determining the temperature-induced unfolded processes.

## Materials and Methods

### Sample Preparation

The recombinant Yfh1 protein was produced as previously described.^33^ In short, the ^15^N-labeled protein was expressed in *Escherichia coli* BL21-(DE3) cells grown at 314 K (37 °C) in minimal medium using ammonium sulfate as the sole source of nitrogen and induced by addition of 0.5 mM IPTG for 2 h. After cell harvesting by centrifugation, the cells were resuspended in Tris–HCl buffer containing a complete EDTA protease inhibitor cocktail tablet (Roche) and lysed by sonication. The soluble protein was purified by two ammonium sulfate precipitation steps at 40% cut to precipitate contaminating proteins and a 65% cut to precipitate Yfh1. The protein was dialyzed and further purified by anion exchange chromatography using a Pharmacia Q-Sepharose column with a gradient from 0 to 1 M NaCl, followed by a Pharmacia phenyl-Sepharose column with a decreasing 1 M ammonium sulfate gradient. The final samples were dialyzed in 20 mM HEPES at pH 7.0. No additional salt was added since we had previously demonstrated that even small quantities of salt appreciably stabilize the protein.^18,20^

### Protein Unfolding Monitored by High-Pressure NMR Spectroscopy

A protein sample with about 0.5 mM concentration of ^15^N-labeled Yfh1 in 20 mM HEPES at pH 7.0, with 2 mM DTT and containing 5% (v/v) D_2_O for the lock, was used on a 5/3 mm O.D./I.D. ceramic tube (330 μL of sample volume) from Daedalus Innovations (Aston, PA, USA). Hydrostatic pressure was applied to the sample directly within the magnet using the Xtreme Syringe Pump, also from Daedalus Innovations. One- ^1^H and two-dimensional [^1^H,^15^N] HSQC spectra were recorded on a Bruker AVANCE III 600 MHz spectrometer (standard ^1^H–^15^N double-resonance BBI probe), in the range 278–303 K, although in the final analysis, we used only those at 283–303 K. At each temperature, the pressure was varied from 1 to 1000 bar by steps of 50 bar. Each pressure step lasted 2 h, starting with 1 h relaxation time, to allow the folding/unfolding reaction to reach full equilibrium, followed by a 1D spectrum (32 scans with 10 s of relaxation time to ensure the complete relaxation of the methyl protons between each scan) and a 2D [^1^H,^15^N] HSQC (8 scans for each of the 128 complex points in the indirect dimension). The relaxation time preceding the recording of the experiments was estimated from a series of 1D NMR experiments recorded after a 300 bar P-Jump, following the exponential growth of the resonance band corresponding to the methyl groups in the unfolded state of the protein (Dubois et al., 2020^40^). Reversibility of unfolding was checked by comparing 1D and 2D [^1^H,^15^N] HSQC spectra recorded at the end of the series of experiments after returning at 1 bar with the spectra recorded at 1 bar before pressurization.

^1^H chemical shifts were directly referenced to the methyl resonance of DSS (2,2-dimethyl-2-silapentane-5-sulfonate, sodium salt), while ^15^N chemical shifts were referenced indirectly to the absolute frequency ratios ^15^N/^1^H = 0.101329118. The water signal was suppressed by the WATERGATE pulse sequence.^34^ The assignment of the amide resonances of Yfh1 was retrieved from the BioMagResBank (http://www.bmrb.wisc.edu, entry number 19991). Data processing and analysis of the HSQC experiments were performed using GIFA.^35^ At each temperature, the cross-peak intensities for the folded species were measured at each pressure and then fitted with a sigmoidal curve characteristic of a two-state equilibrium:
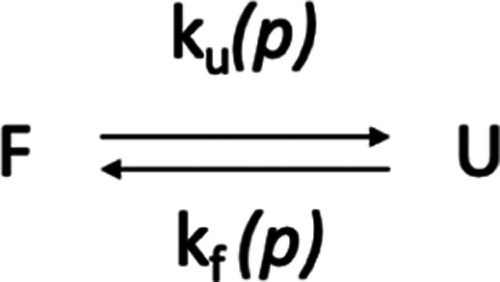
where *F* and *U* correspond to a given residue (identified through its amide cross-peak) sitting in a folded or unfolded state, respectively, in equilibrium with an equilibrium constant *K*_eq_ of
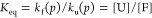
1where *k*_f_(*p*) and *k*_u_(*p*) stand for the folding and unfolding rate constants at a given pressure *p*. *K*_eq_ can also be expressed by the Boltzmann equation as
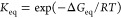
2Assuming a two-state folding reaction, the derivative of Δ*G*_eq_ with respect to temperature and pressure *d*(Δ*G*_eq_) = −ΔSd*T* + Δ*V*d*p* can be integrated as a second-order Taylor series expansion around (*p*_0_, *T*_0_) reference points.^36^ When considering the terms varying only for pressure (constant temperature), Δ*G*_eq_ becomes
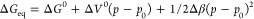
3Δ*G*_eq_ and Δ*G*^0^ are the Gibbs-free energy changes from *F* to *U* at pressure *p* and *p*_0_ (*p*_0_ = 1 bar), respectively; Δ*V*^0^ is the variation of partial molar volume; Δβ is the variation in the compressibility coefficient, *R* is the gas constant, and *T* is the absolute temperature. It has been shown that, for proteins, the difference in compressibility between native and denatured states is negligible.^37^ Thus, the expression of Δ*G*_eq_ simplifies to
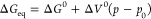
4

Using amide cross-peak intensities *I* as the observables, the equilibrium constant can be written as
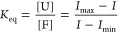
5where, for each temperature, *I* is the cross-peak intensity^38^ of the folded species measured at a given variable pressure, and *I*_max_ and *I*_min_ correspond, respectively, to the intensities of the same cross-peak in the fully native (low pressure) and in the unfolded states (high pressure). Note that the use of cross-peak volumes could give a better relative estimation of the folded/unfolded populations since volumes take into account any pressure-dependent line-broadening arising from relaxation and water exchange. Nevertheless, accurate measurement of peak volume is limited to nonoverlapping peaks. In cases of partial overlap, the use of complex and sometimes cumbersome deconvolution methods can lead to underestimating or overestimating the real value of the peak volumes. This could lead to discarding all overlapping peaks from the analysis. This is of course not the case when peak intensities are used, which can be obtained with higher accuracy also in the case of partial overlapping. We have anyway previously shown that, within the pressure range used for our experiments, the above-mentioned effects affecting the line-broadening during pressurization are largely negligible as compared to the intensity decrease due to denaturation.^38^ We have also compared the results using volumes and intensities for a representative subset of peaks, observing a qualitative excellent agreement (data not shown). Combining eq 5 with eqs 2 and 4 gives the characteristic equation for a two-state equilibrium
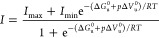
6In the fitting, *I*_max_, *I*_min_, Δ*G*_u_^0^, and Δ*V*_u_^0^ values were left as floating parameters. Once the Δ*G*_u_^0^ and Δ*V*_u_^0^ values were obtained, the residue-specific curves were normalized (*I*_max_ = 1 and *I*_min_ = 0 in eq 6) to give the native fraction of the protein as a function of pressure. Errors on the cross-peak intensities were estimated from the noise level, measured on an empty zone of the 2D HSQC spectra (“baseline” module in GIFA). They were further used to calculate the errors on Δ*G*_u_^0^ and Δ*V*_u_^0^ with a Monte Carlo procedure.

### Calculation of the Δ*H*_m_, Δ*C*_p_, and *T*_m_ Values at Ambient Pressure

Assuming that the difference in heat capacity, Δ*C*_p_, between native and unfolded state is temperature-independent, Δ*G*^0^ at constant pressure depends on temperature as described by the modified Gibbs–Helmholtz equation
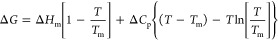
7where *T*_m_ is the temperature at the midpoint of the unfolding transition and Δ*H*_m_ is the unfolding enthalpy change at *T*_m_. The curve corresponding to this equation is known as the stability curve of the protein. The thermodynamic parameters Δ*H*_m_, Δ*C*_p_, and *T*_m_ were obtained by fitting to eq 7 the average values ⟨Δ*G*_u_^0^⟩ obtained by averaging the residue-specific values of Δ*G*_u_^0^ measured at each temperature for each residue, through their corresponding residue-specific pressure denaturation curve. Nonlinear fitting of equations by experimental data was carried out using the Levenberg–Marquardt algorithm.

### Calculation of the Contact Maps

According to a method previously developed,^24^ we defined the probability of contact for each pair of residues, *P*_*i*,*j*_, as the geometric mean of the fractional probability of the two residues at a given pressure using the relation^39^
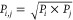
8where the fractional probability *P*_*i*_ or *P*_*j*_ correspond to the probability to find residue *i* or *j* in the native state at a given pressure for a given temperature. These fractional probabilities are obtained directly from the normalized residue-specific denaturation curves obtained for each residue.^40^ Using CMview^41^ (http://www.bioinformatics.org/cmview/) with a generous cutoff distance threshold of 9.5 Å, we then plotted the evolution of the number of lost contacts as a function of pressure and temperature, assuming that a contact is “lost” when *P*_*i,j*_ < 0.5. We repeated the analysis both on an AlphaFold model obtained by the AlphaFold2.2 software^42^ and on the NMR structure (2ga5) finding comparable results.

## Results

### Yfh1 Undergoes Pressure Denaturation under Modest Pressures

To get residue-specific information on the unfolding of Yfh1, we recorded 1D ^1^H and 2D [^1^H,^15^N] HSQC spectra in the pressure range of 1–1000 bar and between 278 and 303 K with a 5° interval to analyze Yfh1 pressure unfolding at the residue level.^27,40,43^ This range of pressure was chosen because previous high-pressure fluorescence spectroscopy studies of Yfh1 had demonstrated that the protein has the midpoint of unfolding between 100 and 200 bar.^16^ Note that the spectra at 278 K were recorded but not further analyzed: at this temperature, the protein is already largely unfolded at atmospheric pressure and the remaining resonances corresponding to the native fraction are severely broadened. For reference, we also compared the behavior of the 1D peak areas versus temperature at atmospheric pressure with previous similar plots^17^ to assess reproducibility and observed a good qualitative agreement within experimental errors (Figure S1).

**Figure 2:**
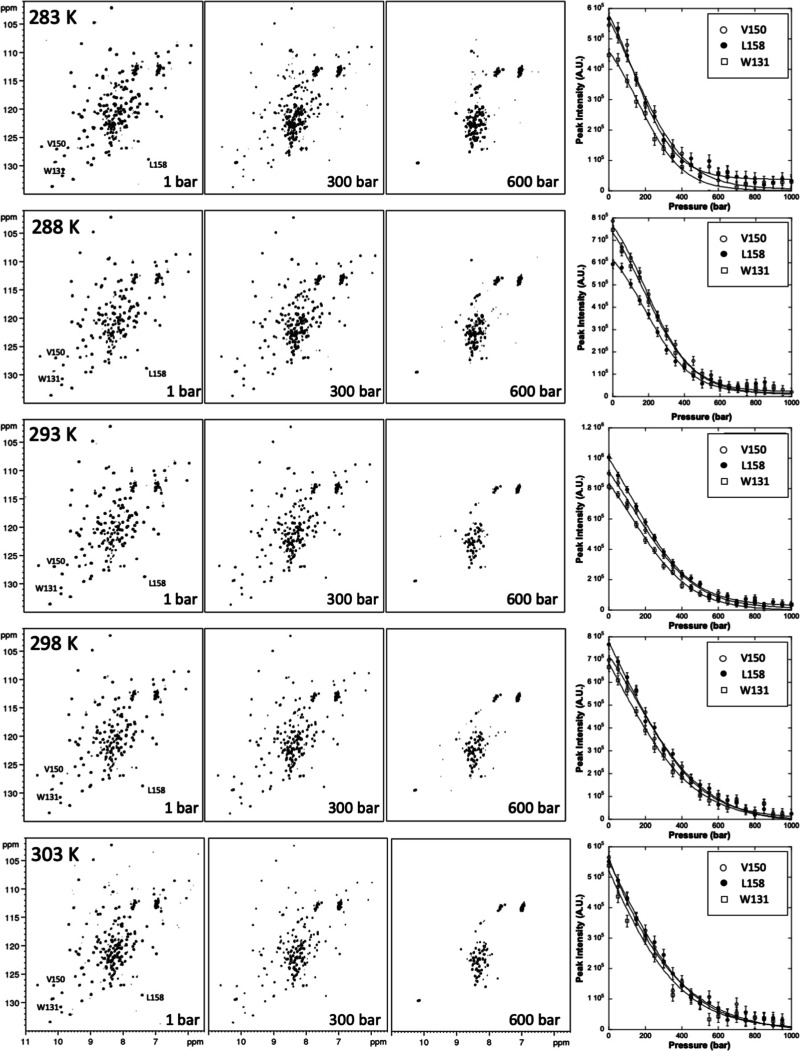
NMR monitored high-pressure unfolding of Yfh1. [^1^H,^15^N] HSQC spectra recorded at 283, 288, 293, 298, and 303 K (from top to bottom, as indicated). At each temperature, spectra at 1, 300, and 600 bar are displayed from left to right. The rightmost panels report the overlays of residue-specific denaturation curves for three representative residues (I131, V150, and L158, labeled on the corresponding HSQC at 1 bar) obtained from the fits of the pressure-dependent sigmoidal decrease of the corresponding residue cross-peak intensities in the HSQC spectra with eq 6.

The 2D [^1^H,^15^N] HSQC spectra at 1 bar, recorded at different temperatures and low ionic strength, were of excellent quality, with well-dispersed resonances and several high- and low-frequency resonances, which demonstrated that the protein is mainly folded in the range of temperatures 283–303 K (Figure 2). As is usually observed, the spectra at increasing pressures showed progressive attenuation of the resonances from the folded species. Conversely, the resonances of the unfolded species became increasingly intense, and peaks originally less intense or masked by other more intense resonances appeared. This behavior indicated the presence of an equilibrium between folded and unfolded species in a slow exchange regime in the NMR time scale. An example is the cross-peak at 10.1 ppm/129.5 ppm (^1^H/^15^N) that corresponds to the indole resonances of the two Trp residues in the unfolded species (Figure S2). These resonances that are present also in the spectrum at 1 bar and at the temperature of maximal stability disappear upon addition of also minute concentrations of salt.^18^ This simple model can be used to interpret the loss of intensity for each native state cross peak, even though the global protein unfolding does not likely conform to a two-state transition, locally.

A total of 67 resonances (corresponding to 59% of the 114 nonproline residues) had no overlap with other peaks at any of the five temperatures and displayed cross-peaks of sufficient intensity at atmospheric pressure to be accurately fit by a two-state model, providing an appreciable number of local probes for the description of Yfh1 unfolding. Above ca. 400 bar, we observed the nearly complete disappearance of the well-dispersed species, suggesting almost complete unfolding of the protein. Some residual structure remained visible also at 600 bar at the noise level, but the relative ratio accounts for less than 2% of the initial intensity. The high-pressure spectra of the pressure-unfolded species exhibited a similar collapse of the resonances at all recorded temperatures, indicating that the primary denaturing agent is pressure (Figure 2).

After reaching 1000 bar, the sample was returned to 1 bar in two steps (500 and 1 bar). The spectra collected before and after pressurization were superimposable within 10% difference, demonstrating the almost complete reversibility of the process. This observation is fully in line with our previous experience with Yfh1, which is a protein altogether with relatively low tendency to aggregate (covalently and noncovalently) also under extreme conditions.

These results qualify Yfh1 as a natural protein unusually sensitive to pressure that unfolds reversibly at modest pressures as compared to those needed for other globular proteins (e.g.,refs (23 and 24)).

### Cold and Pressure Unfolding States Share Closer Features

In previous studies, we had made a detailed comparison between the chemical shifts of the amide protons of the unfolded states at low and high temperatures.^44,45^ We had found that the amide secondary chemical shifts, that is the difference between the experimental values from a given residue and the random-coil values recorded at 298 K for model tetrapeptides,^46^ had consistent different and opposite signs: the values measured at low temperature were negative, implying deshielding, whereas those at high temperature were positive, indicating shielding effects.^44^ We explained these results by a different degree of hydrogen bonding of the amides with water, reflecting a higher degree of hydration at low temperature.

**Figure 3:**
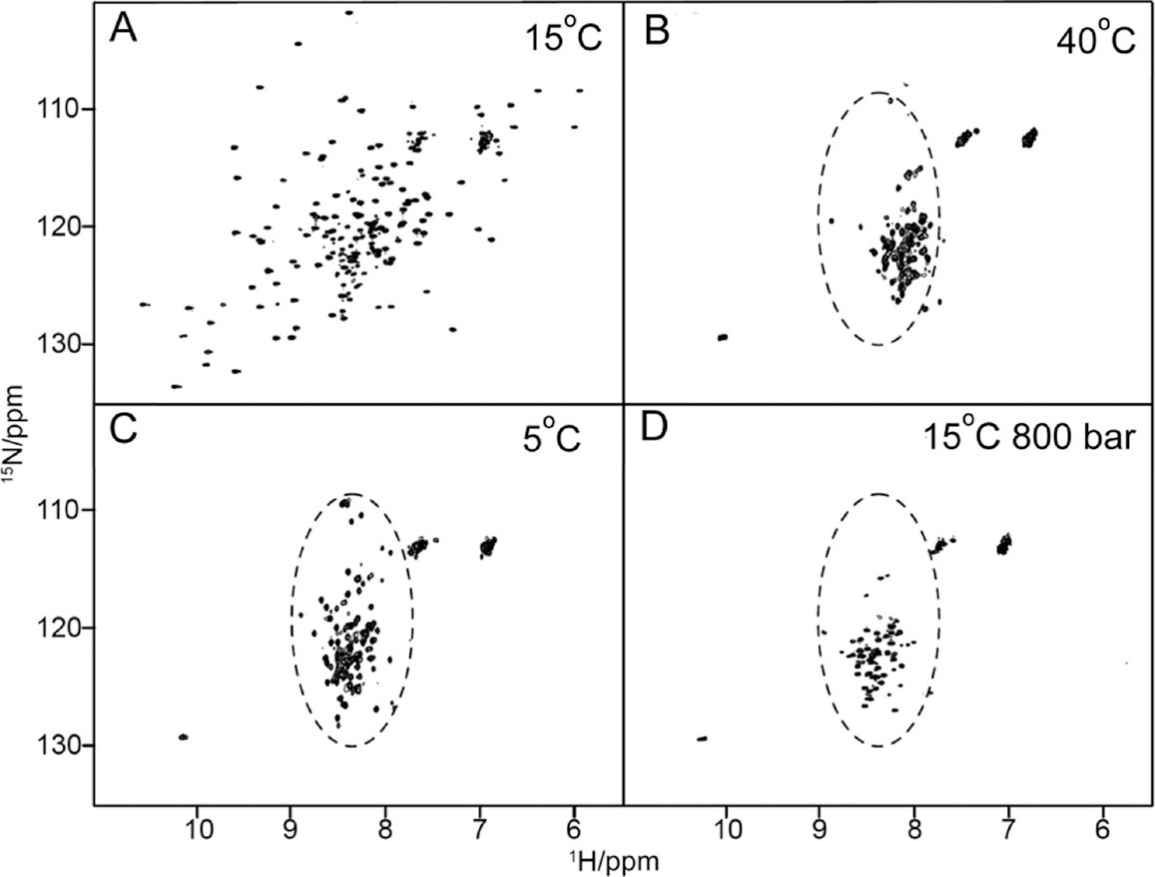
Comparison of the HSQC spectra of Yfh1 under different conditions. (A) Spectrum recorded at the temperature of maximal stability of the protein and room pressure. The spectrum has excellent dispersion and all the features typical of a folded protein. (B) Unfolded spectrum at 313 K and room pressure. (C) Unfolded spectrum at 278 K and room pressure. (D) Unfolded spectrum at 288 K and 800 bar. A dotted oval is drawn in the same region of the various unfolded spectra, which roughly corresponds to the area span by all peaks in the cold denatured spectrum. The three spectra show a similar collapse of the resonance dispersion, but the heat denatured is noticeably up-shifted as compared to the cold denatured spectrum as previously analyzed (Adrover et al., 2010^44^ and 2012^45^). This indicates a different level of hydration that is higher at low temperature. The pressure-denatured spectrum has overall features closer to the cold denatured one.

Here, we compared the spectra of the unfolded states at 15 °C, where the protein reaches its maximal stability, and at high pressure (800 bar) with those obtained at high and low temperatures and atmospheric pressure.^44^ (Figure 3). Several observations could be extracted from the comparison: first, a similar level of collapse of the spectrum is evident in all three conditions, indicating a population of mostly unfolded states. Second, the spectrum at high pressure looks overall closer to that at low temperature and 1 bar, while the high-temperature spectrum appears to be more broadened, likely indicating a higher degree of conformational exchange between species. Third, the spectrum at high pressure exhibits an overall low-field shift, which suggests a deshielding similar to that at low temperature and 1 bar. This can be seen by comparing the overall position of the spectra to an ideal oval plotted in the same position. The averaged value of the proton chemical shifts is in fact 8.35 ppm for the cold and for the high pressure spectra, to be compared with 8.15 ppm for the high temperature unfolded state. The values for the respective nitrogens are less sensitive and are comparable. The shift is also evident for the well-isolated resonances of the only two tryptophan residues in the sequence, one buried and one exposed, that are visible around 10.2 and 129 ppm in the proton and nitrogen dimensions, respectively, in all denatured spectra (Figure 3). Due to sequential effects, the two peaks are not completely superimposable, indicating a residual local level of asymmetric environments, but they are clearly downfield shifted in the cold denatured and high-pressure spectra by ca. 0.2 ppm as compared to high temperature.

Altogether, these observations can be explained by a similar degree of hydrogen bonding strength of the amides with water at low temperature and high pressure, indicating a higher similarity between these two unfolded states compared to the high temperature one.

### Exploring the Unfolding Pathways of Yfh1 at Different Temperatures by High-Pressure NMR

**Figure 4:**
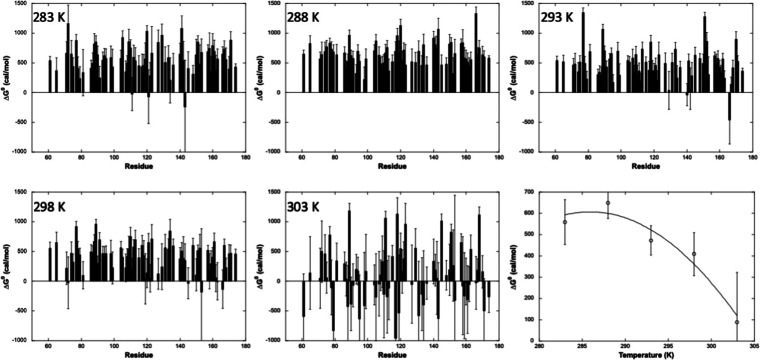
Apparent residue-specific difference of Gibbs free energy of unfolding (Δ*G*_u_^0^) values measured for Yfh1 at 283, 288, 293, 298, and 303 K (as indicated) from residue-specific pressure denaturation curves. The residue numbering used in the figure corresponds to the one deposited for the X-ray structure (3eoq). The last panel displays the averaged values of Δ*G*_u_^0^ at each temperature versus the temperature, fitted with the Gibbs–Helmholtz equation (eq 7).

**Figure 5:**
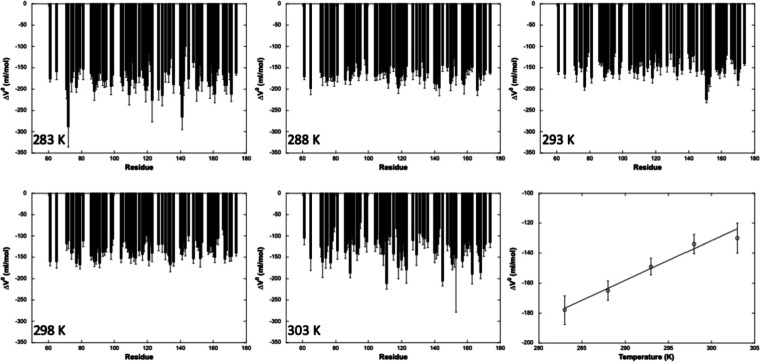
Apparent residue-specific volume variations of unfolding (Δ*V*_u_^0^) values measured for Yfh1 at 283, 288, 293, 298, and 303 K (as indicated) from residue-specific pressure denaturation curves. The residue numbering used in the figure corresponds to the one deposited for the X-ray structure (3eoq). The last panel displays the linear fit of the averaged values of Δ*V*_u_^0^ at each temperature versus the temperature.

Fitting the cross-peak intensities with the corresponding two-state eq (eq 6) provided residue-specific values for the apparent free energy (Δ*G*_u_^0^) of unfolding (Figure 4), which reports on the protein stability, and the apparent volume change (Δ*V*_u_^0^) of unfolding (Figure 5), which corresponds to the volume difference between the folded and unfolded states of the protein.

The two-state model was adequate to fit most of the residue-specific unfolding curves, even though the absence of points describing the upper plateau (*I*_max_) of the sigmoid, due to partial unfolding already at 1 bar, yielded significant error bars for the apparent residue-specific free energy values Δ*G*_u_^0^ and, to a lesser extent, for the residue-specific Δ*V*_u_^0^ values (Figures 4 and 5). This is especially true for experiments recorded at 303 K, a temperature at which several residues exhibited negative values of Δ*G*_u_^0^, meaning that they are in an unfolded conformation at atmospheric pressure for more than half of the protein population.

Yfh1 displays low stability that appears to be maximal around 288 K, with an average value for the apparent free energy of unfolding ⟨Δ*G*_u_^0^⟩ of 649 ± 145 cal/mol, and significantly decreases at higher and lower temperatures (Table 1). These values are remarkably low when compared to what usually observed for other small globular proteins,^4^ which often needed the presence of guanidinium chloride to observe pressure unfolding within a feasible pressure range.^23,24,47,48^ The dependence of ⟨Δ*G*_u_^0^⟩ on temperature exhibited a concave profile with a maximum around 288 K (Figure 4). Fitting the temperature dependence of ⟨Δ*G*_u_^0^⟩ at atmospheric pressure to the Gibbs–Helmholtz equation (eq 7) yielded the averaged thermodynamic parameters Δ*C*_p_ of unfolding (1 ± 0.7 kcal/mol K), *T*_m_ (305 ± 4 K), and Δ*H*_m_, (19 ± 7 kcal/mol) (Figure 4 and Table 2), which are in excellent agreement with previous thermal denaturation studies.^17,49^

**Table 1:** Average Residue-Specific Apparent Free Energy (⟨Δ*G*_u_^0^⟩) and Volume (Δ*V*_u_^0^) of Unfolding Values Measured at Equilibrium and at Atmospheric Pressure and Different Temperatures^a^

temp (°C/K)	present study (NMR)		Puglisi et al.,^16^ 2022 (fluorescence)	
	(kcal/mol)	(mL/mol)	(kcal/mol)	(mL/mol)
5/278	ND	ND	–0.20 ± 0.02	–87 ± 2
10/283	0.559 ± 0.212	–178 ± 19	–0.03 ± 0.02	–90 ± 4
15/288	0.649 ± 0.145	–165 ± 13	0.09 ± 0.05	–90 ± 2
20/293	0.472 ± 0.138	–149 ± 11	0.22 ± 0.07	–83 ± 3
25/298	0.409 ± 0.202	–134 ± 13	0.17 ± 0.02	–81 ± 2
30/303	0.089 ± 0.264	–130 ± 20	–0.08 ± 0.02	–70 ± 1
40/313	ND	ND	–0.19 ± 0.05	–59 ± 1

a The values are compared to the equivalent ones obtained from fluorescence spectroscopy (Puglisi et al.,^16^ 2022).

**Table 2:** Comparison of the Thermodynamic Parameters for Cold and Heat Unfolding of Yfh1 at Atmospheric Pressure Obtained in the Present Study with Values Previously Reported

	Δ*H*_m_ (kcal mol^–1^)	Δ*C*_p_ (kcal K^–1^ mol^–1^)	*T*_c_ (°C)	*T*_m_ (°C)
present study	19 ± 7	1.0 ± 0.7	n.d.	305 ± 4
Pastore et al.,^17^ 2007	21 ± 2	1.8 ± 0.1	7.0 ± 1	304 ± 2
Martin et al.,^49^ 2008	20 ± 2	1.9 ± 0.2	8.0 ± 1	302 ± 2

A linear decrease with the temperature was observed for the average absolute values of the apparent Δ*V*_u_^0^ (Figure 5). The slope of this dependence of ⟨Δ*V*_u_^0^⟩ on temperature corresponds to the difference in thermal expansivity, Δα, between the folded and unfolded states of the protein. The estimated value of Δα = 2.7 ± 0.7 mL/mol.K is comparable to those found in the literature.^39,50−53^ On the other hand, the absolute values of the individual Δ*V*_u_^0^ (≈150 mL/mol) are substantially greater than those usually found for proteins of comparable size and from those measured on Yfh1 by fluorescence spectroscopy (≈90 mL/mol).^16^

**Figure 6:**
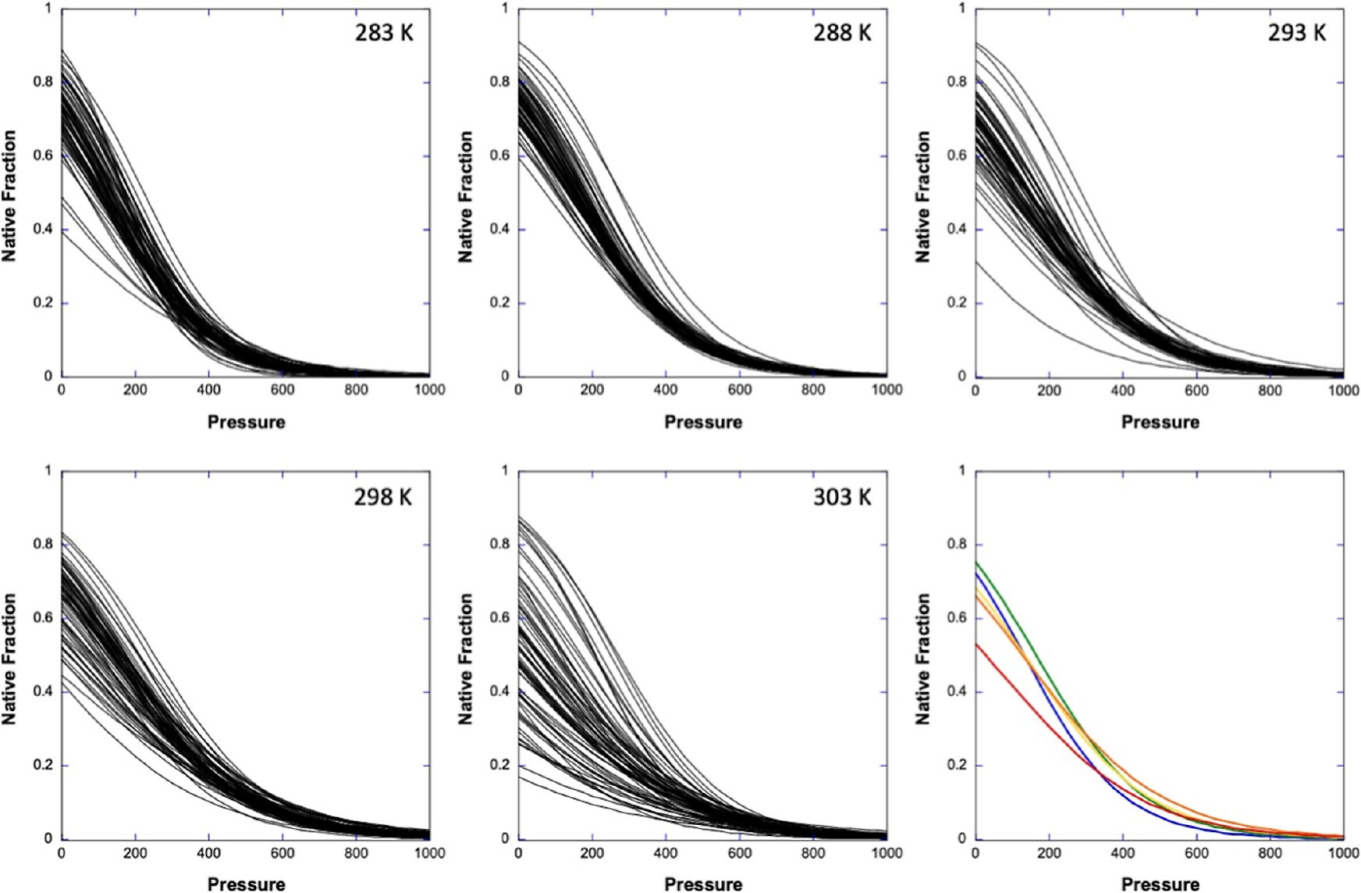
Normalized residue-specific denaturation curves obtained at different temperatures. The last panel displays the overlay of the average normalized curves calculated at 283 K (blue), 288 K (green), 293 K (yellow), 298 K (orange), and 303 K (red).

The residue-specific apparent values of Δ*V*_u_^0^ and Δ*G*_u_^0^ were used to build normalized residue-specific denaturation curves, giving the fraction of folded species for each residue as a function of pressure for each of the five temperatures used in this study (Figure 6).^24,40^ The average normalized curve was then calculated from the individual residue-specific normalized curves, giving information about the global evolution of the native fraction of the protein during pressure unfolding. Although the native fraction reaches 0 around 800 bar at all temperatures, the corresponding values at room atmosphere differ: a maximum value of 0.75 is observed at 15 °C, the temperature of maximal stability, but the values decrease at lower (0.72 at 10 °C) and higher (0.53 at 30 °C) temperatures.

### Evidence of Different Unfolding Pathways at Different Temperatures

As already reported for other proteins,^24^ it is possible to characterize the folding pathway of a protein by mapping on its structure the regions that progressively become unfolded at increasing pressures. In this approach, the probability of contact for each pair of residues *i* and *j*, *P*_*i,j*_, is given by a function of the fractional probabilities obtained, at a given temperature, from the normalized residue-specific denaturation curves for each residue that are, in turn, a function of the peak intensities of the two peaks (ref (40), see Materials and Methods for details). In the following, we considered that a contact between two residues *i* and *j* is lost when *P*_*i,j*_ < 0.5.

Consistent with the Δ*G*_u_^0^ values, the number of lost contacts at 150 bar was much less at 288 K than at lower or higher temperatures, reflecting the higher stability of the protein at this temperature (Figure S3). Virtually all contacts were lost at 200 bar at all temperatures, with partial unfolding of the protein starting at 50 bar at 283, 293, and 298 K. Note that at this pressure, the number of lost contacts is above 60% at 303 K, confirming that the protein is severely denatured under these conditions. At 288 K, the unfolding transition appears more cooperative, with only 6% of the lost contacts at 125 bar. This strongly suggests the important observation that temperature affects not only the stability of the protein but also the cooperativity of unfolding.

We then compared the contact losses at different pressures with the contact map obtained from the 3D structure of Yfh1. A protein contact map represents the distance between all possible pairs of amino acids in a three-dimensional structure using a two-dimensional binary matrix: for two residues *i* and *j*, the element of the matrix *i*,*j* is 1 if the two residues are closer than a predetermined threshold, or 0 otherwise. The map contains all of the information relative to the three-dimensional structure but in a more compact way.

Although the structures of several Yfh1 orthologues from different species have been solved experimentally, the structure of the yeast protein remains determined only at low resolution: the NMR structure (2ga5) has a poor geometry, whereas the X-ray structure corresponds to a mutant that results in a shorter N-terminal helix. For this reason, we obtained an AlphaFold model that recapitulates all the features expected for Yfh1 but with a better geometry (for details see Supporting Information and Figure S4) and used this as a reference structure.

**Figure 7:**
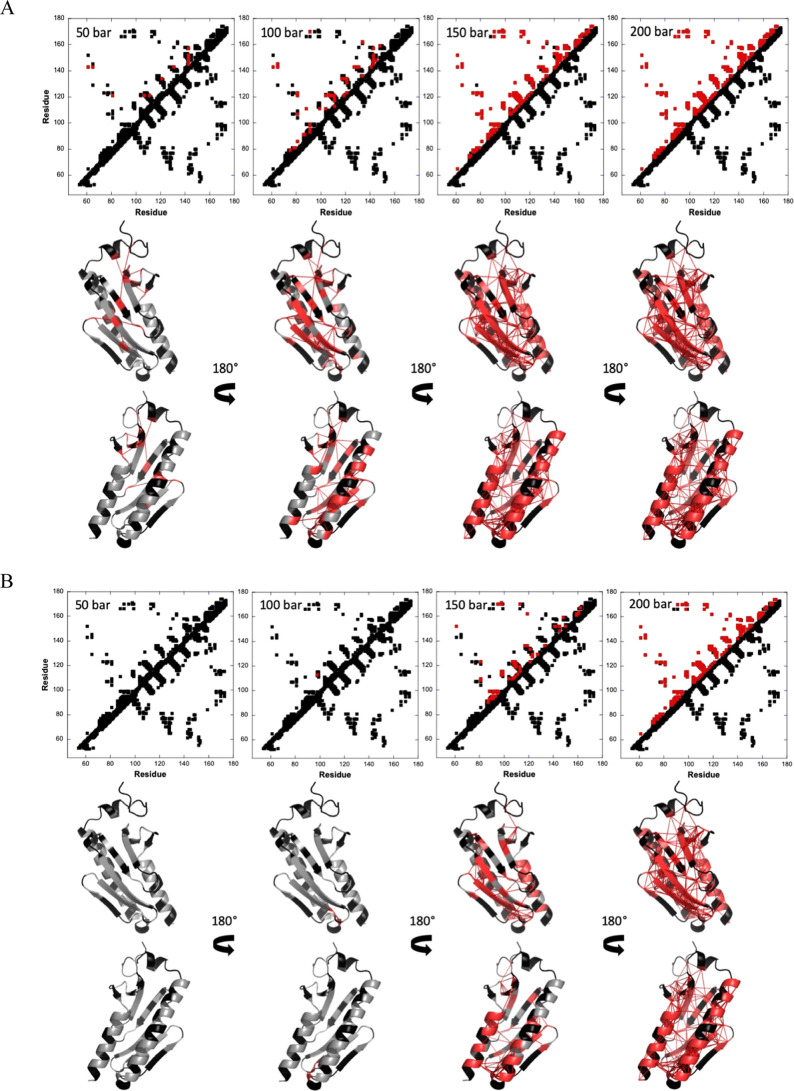

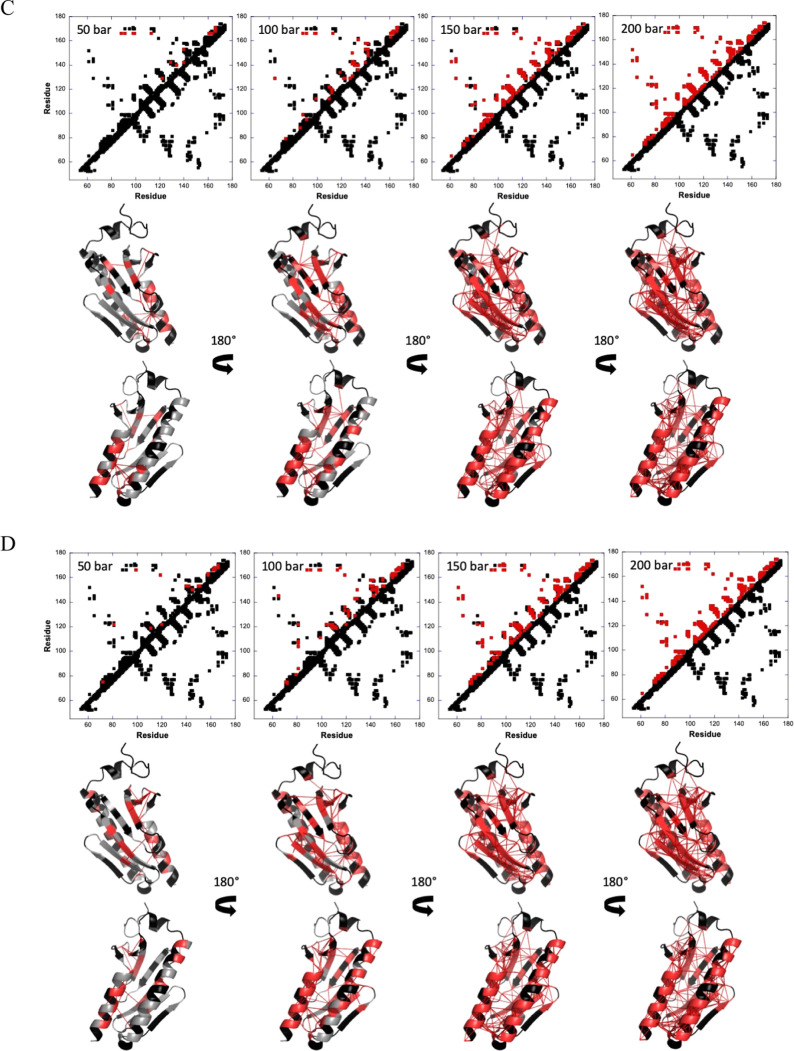

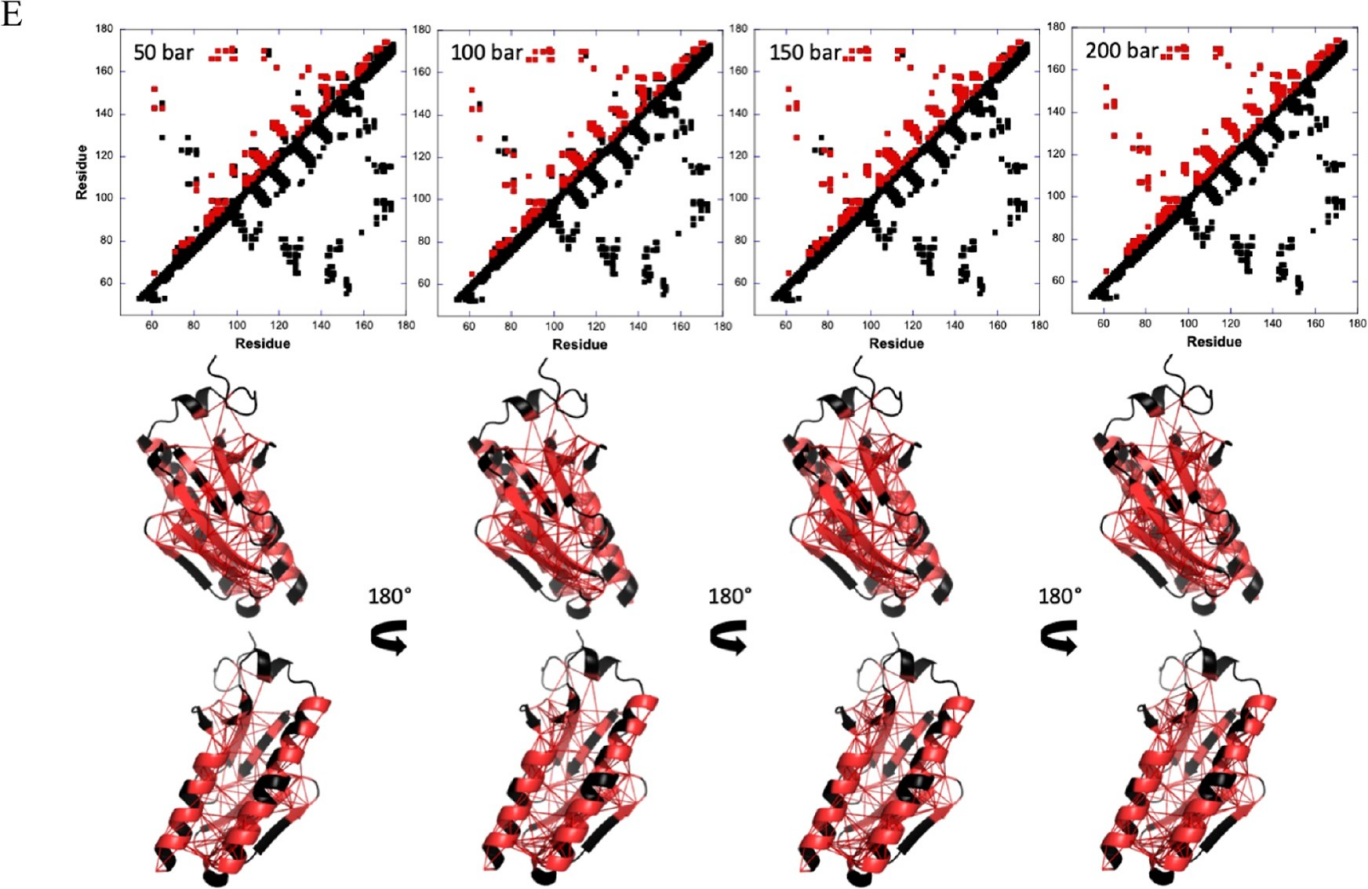
The pathway of pressure denaturation of Yfh1 at different temperatures and pressure. Data collected at (A) 283, (B) 288, (C) 293, (D) 298, and (E) 303 K. Top: comparison between the protein contact map and the information provided by the experiments at different temperatures and pressures. Contact maps are built by plotting the contacts between residues along the two axes of a graph reporting residue number vs residue number. These contacts were defined through the measurement of distances between Cα atoms in the 3D protein structure: below a given threshold (here 9.5 Å), the residues are supposed to be in contact. A contact map contains all the information contained in the 3D structure but in a more compact way. In the figure, the contacts between residues i and j in the 3D structure of Yfh1 are plotted below the diagonal. Above the diagonal, only the contacts between residues for which a specific denaturation curve was measured by high-pressure NMR data are reported. Once normalized, the values on the denaturation curve are expected to vary between 1 (at low pressure, on the upper plateau of the sigmoid) and 0 (at high pressure, lower plateau of the sigmoid). At a given pressure, this value corresponds to the “fractional probability” for a given residue, meaning its probability to sit in a folded environment at a given pressure (*P*_*i*_ = 1 for a residue sensing a local fully folded environment, *P*_*i*_ = 0 for a residue sensing a fully unfolded environment). From these fractional probabilities, it is possible to calculate the probability of contact (*P*_*i*,*j*_) between residues *i* and *j* at a given pressure through eq 8. In the figure, all contacts with a probability of contact ≤ 0.5 (corresponding to a ≪local≫ half-denaturation) were calculated and colored in red. The appearance of a red spot at a given pressure thus means that the protein has “lost” that contact. This allows reconstruction of the regions that unfold first at each pressure. Bottom: visualization of the probabilities of contact on ribbon representations of Yfh1 (with opposite views that differ for a 180° rotation along the vertical axis) at 50, 100, 150, and 200 bar. The red lines represent contacts that are significantly weakened (*P*_*ij*_ < 0.5) at the corresponding pressure. Residues involved in these contacts are colored in red; residues for which fractional probability cannot be obtained are colored in black. Notice that qualitatively comparable results were obtained using the experimental 2ga5 structure (data not shown).

We then built fractional contact maps from the probabilities of contact calculated at the five temperatures and compared them with the contact map calculated from the reference structure (Figure 7). Using a Cα–Cα distance threshold of 9.5 Å, a total of 662 (nonsequential) contacts between the Cα of the 123 residues of the protein could be measured from the AlphaFold model of Yfh1. Of them, 250 contacts concern the 67 residues for which residue-specific denaturation curves could be obtained. Analysis using the 2ga5 structure resulted in qualitatively comparable results (data not shown), demonstrating that this simple but cunning approach is relatively insensitive to the resolution of the reference structure.

At 283 K, few long-range contacts are lost (*P*_*i,j*_ < 0.5) in the β-sheet at 50 bar, a few long-range contacts in the β-sheet, and a few long-range contacts between the beginning of the β-sheet and the N-terminal helix. At 100 bar, contact loss concerns more residues in the β-sheet and also long-range contacts between the β-sheet and the two helices. At this pressure, the N-terminal helix begins to unfold, whereas the C-terminal helix remains mostly unaffected. At 150 bar, almost all contacts are lost. At 288 K, which is close to the maximal stability temperature, unfolding appears very cooperative, starting only at 150 bar, and concerning all the secondary structure elements, as well as the tertiary contacts between them. At 200 bar, virtually all contacts are lost, confirming the higher cooperativity of the unfolding reaction at this temperature. At higher temperatures, contrary to what was observed at 283 K, unfolding becomes again less cooperative, and contact loss concerns first the C-terminal helix (50 bar) and then extends to the β-sheet (100 bar), the N-terminal helix being the last secondary structure element to unfold. At 303 K, almost all contacts are already lost at 50 bar, hampering comparison with the other temperatures.

These results indicate that temperature not only modifies the onset and the cooperativity of the unfolding transition but also affects the unfolding pathway. This important observation suggests a different mechanism of unfolding for the high- and low-temperature transitions that is revealed at low pressure values, allowing us to follow the early stages of unfolding.

## Discussion

Here, we report a study of the properties under pressure-induced unfolding of Yfh1, a small globular yeast protein, and a member of the frataxin family highly conserved from bacteria to primates.^54^ Pressure denaturation studies are interesting because they allow us to reach an important part of the phase diagram of protein unfolding that cannot be accessed otherwise. Yfh1 is a unique model system that, thanks to its marginal stability, is particularly suited to study the mechanisms of protein unfolding under different denaturing agents.^22,26^ As compared to other studies carried out to understand pressure unfolding using stable proteins, the marginal stability of Yfh1 allows us to unfold completely the protein under mild perturbations without the addition of chaotropic agent such as urea or guanidinium that, inherently, perturb the chemical environment and changes the solvation state of proteins,^26^ and compare directly the unfolded states resulting from cold, heat, and high-pressure unfolding.

In a previous study based on the intrinsic fluorescence of the two tryptophan residues of Yfh1, we had used pressure as a means of structural perturbation to obtain information on the global response of Yfh1 and describe the phase diagram of the protein.^16^ We had shown that, as expected from its marginal stability, Yfh1 can be pressure-unfolded at values appreciably lower than those required for unfolding most of the small globular proteins reported so far: pressures below ∼600 bar are sufficient to achieve the practically complete unfolding of Yfh1 to be compared to the often >2000 bar needed for other proteins.^4,14^

In the present study, we aimed at gaining information on the pressure unfolding pathways of Yfh1 at the residue-specific level by NMR since this technique is uniquely suited for protein unfolding studies at the residue-specific level, providing local information on the behavior of different regions of a protein, as previously demonstrated in thermal unfolding studies.^55,56^ We recorded spectra in the range of 1–1000 bar and 278–303 K according to previous indications.^16^

We observed a qualitatively similar pattern at all temperatures: according to our previous work,^16^ the unfolding transition starts already at 50 bar. Around 400 bar, the molecule is almost completely unfolded with almost complete but not total disappearance of the well-dispersed resonances from the folded species. Some residual peaks from the folded structure remain also at 600 bar as it can be observed through the retention of resonances from the folded species in the spectrum, but the relative ratio accounts for less than 2% of the initial intensity, which is close to the noise level (data not shown). It is worth mentioning that we had observed the same minor retention of the folded spectrum also in NMR spectra recorded at low and high temperatures but at atmospheric pressure at values at which the CD spectrum of the unfolded species had reached a plateau.^17,45^ This indicates how uniquely sensitive NMR is to detect even minute residual quantities of a species under conditions in which other techniques cannot compete.

Comparison of the thermodynamics parameters obtained from fluorescence and NMR measurements shows that the Δ*G* and Δ*V* values obtained in our prior work^16^ (Table 1) are significantly smaller than those reported here from high-pressure NMR. The discrepancy between the Δ*G* and Δ*V* values could be explained by remembering that fluorescence spectroscopy usually reports on the “global” unfolding of the protein and observes all the states during the unfolding process: unfolded, folded, and potential intermediate states, while, in a slow exchange regime, NMR reflects at a residue level only the folded and unfolded states. Any multiple states populated during unfolding with small Δ*V* would be averaged in fluorescence, resulting in Δ*V* values smaller than those measured by NMR. Accordingly, although fitted assuming a two-states transition as a first approximation, our fluorescence data seem to indicate at least a three-state equilibrium (2 different slopes).^16^ It is also interesting to notice that the thermal expansivity, that is the slope of the Δ*V* as a function of the temperature, is 2.30 mL/mol °C for Yfh1, that is, albeit larger, in the order of magnitude of values reported in other studies for proteins of similar size (e.g., 1.15 mL/mol °C for GIPC10, and 1.71 mL/mol °C for pp32, refs (39 and 50)).

Overall, there are several important and novel conclusions of this study that give us a new perspective of the effects of pressure on protein unfolding. First, our data collectively hint at a higher similarity between the mechanism that governs cold and pressure denaturation over high temperature: the overall clear-cut low-field chemical shifting of the spectra recorded under pressure or at low temperature as compared to the high-temperature spectrum clearly speaks in favor of a deshielding of the amide protons. This conclusion is in excellent agreement with the theoretical study by Dias^31^ who showed that hydrophobic interactions can account for cold and pressure denaturation through formation of solvent-separated configurations, i.e., configurations in which hydrophobic residues are separated from each other by a single layer of water molecules. The authors concluded that the processes of pressure and cold denaturation are driven by the hydration of residues in the nonpolar protein core by a thin layer of water, leaving part of the secondary structures conserved. These conclusions were also supported by a recent study on the effect of pressure on the chemical shifts of the cold shock protein B from *Bacillus subtilis* (*Bs*CspB).^57^ Pressure-assisted cold denaturation using high-pressure quartz NMR tubes^10^ had also hinted at a closer similarity of the pressure-assisted, and cold, and alcohol unfolded states, supporting the notion that, similarly to alcohol, also pressure and cold reduce the hydrophobic effect.^12^ These results are all consistent with Privalov’s theory,^3,58,59^ by which cold denaturation would strongly depend on the higher affinity of water to apolar groups and on hydrogen bonding with the solvent, while heat denaturation is entropically driven, resulting from increasing molecular motions. While our conclusions may have been suggested before, our work is the first study in which experimental data directly indicate a similar role of hydration in cold and pressure unfolding.

Second, our analysis allowed us to follow the pressure, cold, and heat unfolding pathways at the residue level and characterize the different unfolded states at different temperatures helped by pressure-assisted destabilization. Already in 1995, Jonas and co-workers demonstrated the possibility to exploit pressure to assess cold denaturation of RNase A and compare cold, heat, and pressure unfolding states and showed a noncooperative unfolding.^5^ Later on, Babu et al.^60^ and Whitten et al.^61^ demonstrated the presence of a noncooperative ensemble of conformations in the cold but not in the heat-denatured unfolded state of ubiquitin in reverse micelles at atmospheric pressure. This work consolidated the view that proteins, also under native conditions, exist not as a single conformation but as ensembles of interconverting transient microstates. Following studies adopting an ensemble-based model of protein structure, for instance, to characterize the denatured state of a whole database of human proteins,^62^ revealed important sequence-dependent thermodynamic properties of denatured ensembles as well as fundamental differences between the denatured and native ensembles.^61,63^ The possibility to follow ubiquitin under a variety of conditions confirmed that the pressure-assisted cold unfolding of ubiquitin is not a simple two-state process, and that several intermediates exist.^10,12^

Our analyses allowed us to follow the hierarchical mechanism of Yfh1 unfolding. At 288 K, which is close to the maximal stability, pressure unfolding is highly cooperative, close to a global two-state equilibrium between the folded and unfolded populations of the protein. Out of this zone of stability, unfolding becomes less cooperative at low pressures, whereas at higher pressures, it is again highly cooperative at all temperatures, indicating a sudden collapse of the structure and the opening of the hydrophobic core.

**Figure 8:**
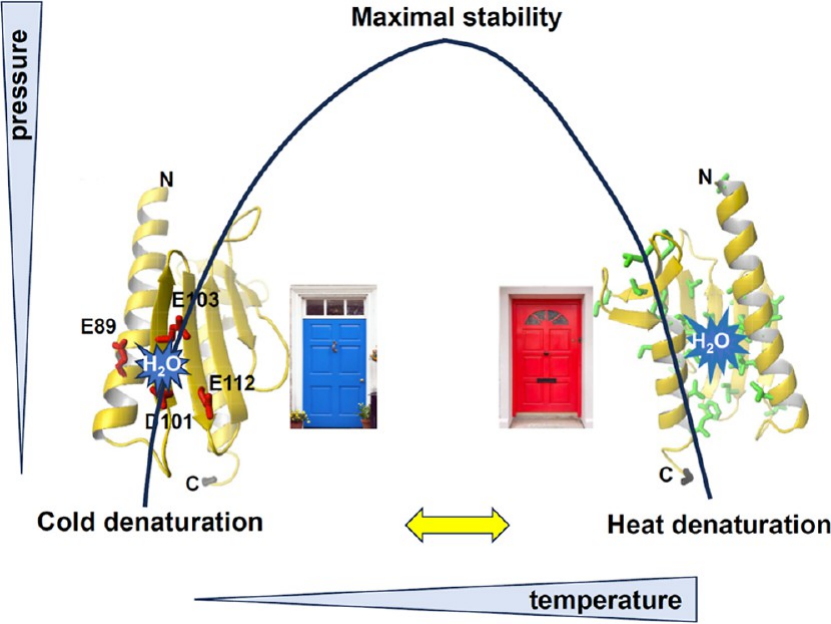
Cartoon representation showing the forces determining Yfh1 unfolding at low and high temperatures under mild pressures. The structure of Yfh1 as shown in Figure 1 is plotted to visualize where water starts entering in the two conditions symbolized as two different virtual doors (indicated in blue and red for cold and heat unfolding): at low temperature, where hydrophobic forces are weaker, the electrostatic repulsion between side chains near the N-terminus of the protein will determine opening of the structure, whereas at high temperature, the partial exposure of the hydrophobic core due to the shorter C-terminus allows the structure to unfold starting from the C-terminus. The process becomes completely cooperative at high pressure and it is no longer possible to distinguish the two pathways.

The data presented here support these previous hypotheses, as we observe directly that the pattern of unfolding at very mildly higher pressures than 1 bar depends on temperature, and we observe two different temperature-dependent unfolding pathways. We could say that, metaphorically speaking, it is as if there were two “doors” on the structure of the protein that promote unfolding, one for the cold temperature, the other for the hot one (Figure 8): at low temperature (283 K), partial unfolding concerns primarily the N-terminal helix and the first strands of the β-sheet, where the negatively charged cluster is.^21,64^ At high temperatures, unfolding affects the C-terminal helix and then extends to the β-sheet. This behavior suggests, for the first time, a synergic mechanism between pressure- and temperature-induced denaturation in which pressure destabilization helps to unveil the hierarchical events of cold- and heat-induced unfolding.

These conclusions should be put into the context of our previous work on the forces contributing to the stability of Yfh1. We have previously shown that the bacterial, yeast, and human orthologues of Yfh1 share the same fold but have very different stabilities, with only the yeast protein being marginally stable.^20^ We proved a strong contribution of the C-terminus of the protein in stabilization: when we cut the much longer C-terminus of the bacterial or human proteins, we drastically reduced the *T*_m_ of these proteins, without affecting the low temperature transition.^20^ When vice versa, we extended the C-terminus of yeast Yfh1, we gained stability (ca. 8 °C in Δ*T*_m_). This is because the C-terminus inserts between the two helices, and without this insertion, the hydrophobic core is more accessible to the solvent. We then discovered that cold denaturation is detectable only for Yfh1 and only at low salt concentrations.^17,65^ We noticed that Yfh1 contains a superficial cluster of spatially close negatively charged residues.^21^ We mutated up to three of these residues to neutral serines and found that these mutations led to some stabilization at high temperature but to a much stronger effect at low temperature, making cold denaturation undetectable. We concluded that electrostatic repulsion between these residues creates a strain that favors opening of the three-dimensional structure under conditions in which hydrophobic forces are weaker and entrance of water in the hydrophobic core, determining unfolding. In support of this hypothesis, we managed to convert the stable bacterial orthologue to a marginally stable protein that undergoes detectable cold denaturation by recreating the electrostatic cluster of the yeast protein through a few mutations.^64^

In summary, our report describes the pressure unfolding of Yfh1 and allowed us to follow the unfolding pathway of the protein as a function of pressure and temperature in a residue-specific way. We could trace the regions of Yfh1 that unfold first and determine a model that could explain the mechanism of unfolding where we have observed an inherent interplay between pressure and temperature. The two main conclusions of our study are the higher similarity between the high pressure and the cold denaturation mechanisms, despite the inherently different physical processes, and directly related to it being the determinant role of hydration in protein stability.

## Supporting Information Available

Analysis of the reference structures and figures showing the dependence of the resonances at room pressure as a function of temperature from 1 and 2D spectra, the indole resonances of the two tryptophan residues of Yfh1 as a function of pressure at 20 °C, the evolution of the loss of contacts as a function of pressure during Yfh1 unfolding, and the comparison of the available structures of Yfh1 (PDF)

## References

[ref1] Schroer M. A.; Paulus M.; Jeworrek C.; Krywka C.; Schmacke S.; Zhai Y.; Wieland D. C.; Sahle C. J.; Chimenti M.; Royer C. A.; Garcia-Moreno B.; Tolan M.; Winter R. High- pressure SAXS study of folded and unfolded ensembles of proteins. Biophys. J. 2010, 99 (10), 3430–3437. 10.1016/j.bpj.2010.09.046. 21081092 PMC2980736

[ref2] Pastore A.; Temussi P. A. The Protein Unfolded State: One, No One and One Hundred Thousand. J. Am. Chem. Soc. 2022, 144 (49), 22352–22357. 10.1021/jacs.2c07696. 36450361 PMC9756289

[ref3] Privalov P. L. Cold denaturation of proteins. Crit. Rev. Biochem. Mol. Biol. 1990, 25, 281–305. 10.3109/10409239009090612. 2225910

[ref4] Smeller L. Pressure-temperature phase diagrams of biomolecules. Biochim. Biophys. Acta 2002, 1595, 11–29. 10.1016/S0167-4838(01)00332-6. 11983384

[ref5] Zhang J.; Peng X.; Jonas A.; Jonas J. NMR study of the cold, heat, and pressure unfolding of ribonuclease A. Biochemistry 1995, 34 (27), 8631–8641. 10.1021/bi00027a012. 7612603

[ref6] Fuentes E. J.; Wand A. J. Local stability and dynamics of apocytochrome b562 examined by the dependence of hydrogen exchange on hydrostatic pressure. Biochemistry 1998, 37 (28), 9877–9883. 10.1021/bi980894o. 9665691

[ref7] Panick G.; Vidugiris G. J.; Malessa R.; Rapp G.; Winter R.; Royer C. A. Exploring the temperature-pressure phase diagram of staphylococcal nuclease. Biochemistry 1999, 38 (13), 4157–4164. 10.1021/bi982608e. 10194332

[ref8] Jacob M. H.; Saudan C.; Holtermann G.; Martin A.; Perl D.; Merbach A. E.; Schmid F. X. Water contributes actively to the rapid crossing of a protein unfolding barrier. J. Mol. Biol. 2002, 318 (3), 837–845. 10.1016/S0022-2836(02)00165-1. 12054827

[ref9] Peterson R. W.; Wand A. J. Self-contained high-pressure cell, apparatus, and procedure for the preparation of encapsulated proteins dissolved in low viscosity fluids for nuclear magnetic resonance spectroscopy. Rev. Sci. Instrum. 2005, 76 (9), 1–7. 10.1063/1.2038087. PMC1343520 16508692

[ref10] Kitahara R.; Okuno A.; Kato M.; Taniguchi Y.; Yokoyama S.; Akasaka K. Cold denaturation of ubiquitin at high pressure. Magn. Reason. Chem. 2006, 44 (S1), S108–S113. 10.1002/mrc.1820. 16826551

[ref11] Fu Y.; Kasinath V.; Moorman V. R.; Nucci N. V.; Hilser V. J.; Wand A. J. Coupled motion in proteins revealed by pressure perturbation. J. Am. Chem. Soc. 2012, 134 (20), 8543–8550. 10.1021/ja3004655. 22452540 PMC3415598

[ref12] Vajpai N.; Nisius L.; Wiktor M.; Grzesiek S. High-pressure NMR reveals close similarity between cold and alcohol protein denaturation in ubiquitin. Proc. Natl. Acad. Sci. U.S.A. 2013, 110 (5), E368–E376. 10.1073/pnas.1212222110. 23284170 PMC3562818

[ref13] Nucci N. V.; Fuglestad B.; Athanasoula E. A.; Wand A. J. Role of cavities and hydration in the pressure unfolding of T4 lysozyme. Proc. Natl. Acad. Sci. U.S.A. 2014, 111 (38), 13846–13851. 10.1073/pnas.1410655111. 25201963 PMC4183293

[ref14] Roche J.; Royer C. A. Lessons from pressure denaturation of proteins. J. R. Soc. Interface. 2018, 15, 20180244 10.1098/rsif.2018.0244. 30282759 PMC6228469

[ref15] Caro J. A.; Valentine K. G.; Cole T. R.; Wand A. J. Pressure, motion, and conformational entropy in molecular recognition by proteins. Biophys Rep 2023, 3 (1), 100098 10.1016/j.bpr.2022.100098. PMC9840116 36647534

[ref16] Puglisi R.; Cioni P.; Gabellieri E.; Presciuttini G.; Pastore A.; Temussi P. A. Heat and cold denaturation of Yeast frataxin: the effect of pressure. Biophys. J. 2022, 121, 1502–1511. 10.1016/j.bpj.2022.03.010. 35278425 PMC9072581

[ref17] Pastore A.; Martin S. R.; Politou A.; Kondapalli K. C.; Stemmler T.; Temussi P. A. Unbiased cold denaturation: low- and high-temperature unfolding of yeast frataxin under physiological conditions. J. Am. Chem. Soc. 2007, 129, 5374–5375. 10.1021/ja0714538. 17411056 PMC2664662

[ref18] Vilanova B.; Sanfelice D.; Martorell G.; Temussi P. A.; Pastore A. Trapping a salt-dependent unfolding intermediate of the marginally stable protein Yfh1. Front. Mol. Biosci. 2014, 1, 1–13. 10.3389/fmolb.2014.00013. 25988154 PMC4428383

[ref19] Musco G.; Stier G.; Kolmerer B.; Adinolfi S.; Martin S.; Frenkiel T.; Gibson T.; Pastore A. Towards a structural understanding of Friedreich’s ataxia: the solution structure of frataxin. Structure 2000, 8, 695–707. 10.1016/S0969-2126(00)00158-1. 10903947

[ref20] Adinolfi S.; Nair M.; Politou A.; Bayer E.; Martin S.; Temussi P. A.; Pastore A. The factors governing the thermal stability of frataxin orthologues: how to increase a protein stability. Biochemistry 2004, 43, 6511–6518. 10.1021/bi036049+. 15157084

[ref21] Sanfelice D.; Morandi E.; Pastore A.; Niccolai N.; Temussi P. A. Cold denaturation unveiled: molecular mechanism of the asymmetric unfolding of yeast Frataxin. ChemPhysChem 2015, 16, 3599–3602. 10.1002/cphc.201500765. 26426928 PMC4676917

[ref22] Temussi P. A.; Martin S. R.; Pastore A. Life and death of Yfh1: how cool is cold denaturation. Q. Rev. Biophys. 2025, 58, e2 10.1017/S0033583524000180. 39801016

[ref23] Herrada I.; Barthe P.; Vanheusden M.; DeGuillen K.; Mammri L.; Delbecq S.; Rico F.; Roumestand C. Monitoring unfolding of titin i27 single and bi domain with high-pressure nmr spectroscopy. Biophys. J. 2018, 115, 341–352. 10.1016/j.bpj.2018.06.010. 30021109 PMC6051020

[ref24] Roche J.; Caro J. A.; Norberto D. R.; Barthe P.; Roumestand C.; Schlessman J. L.; Garcia A. E.; García-Moreno E B.; Royer C. A. Cavities determine the pressure unfolding of proteins. Proc. Natl. Acad. Sci. U.S.A. 2012, 109, 6945–6950. 10.1073/pnas.1200915109. 22496593 PMC3344970

[ref25] Jaworek M. W.; Ruggiero A.; Graziano G.; Winter R.; Vitagliano L. On the extraordinary pressure stability of the Thermotoga maritima arginine binding protein and its folded fragments - a high-pressure FTIR spectroscopy study. Phys. Chem. Chem. Phys. 2020, 22 (20), 11244–11248. 10.1039/D0CP01618G. 32400824

[ref26] Pastore A.; Temussi P. A. Unfolding under pressure: an NMR perspective. ChemBioChem 2023, 24, e202300164 10.1002/cbic.202300164. 37154795

[ref27] Roche J.; Royer C. A.; Roumestand C. Monitoring protein folding through high pressure NMR Spectroscopy. Prog. Nucl. Magn. Reson. Spectrosc. 2017, 102–103, 15–31. 10.1016/j.pnmrs.2017.05.003. 29157491

[ref28] de Oliveira G. A.; Silva J. L. A hypothesis to reconcile the physical and chemical unfolding of proteins. Proc. Natl. Acad. Sci. U S A 2015, 112 (21), E2775–E2784. 10.1073/pnas.1500352112. 25964355 PMC4450381

[ref29] de Oliveira G. A. P.; Arruda H. R. S.; de Andrade G. C.; Silva J. L. Evolutionary Role of Water-Accessible Cavities in Src Homology 2 (SH2) Domains. J. Phys. Chem. B 2022, 126 (43), 8689–8698. 10.1021/acs.jpcb.2c05409. 36281877

[ref30] Dias C. L.; Ala-Nissila T.; Karttunen M.; Vattulainen I.; Grant M. Microscopic mechanism for cold denaturation. Phys. Rev. Lett. 2008, 100 (11), 118101 10.1103/PhysRevLett.100.118101. 18517830

[ref31] Dias C. L. Unifying microscopic mechanism for pressure and cold denaturations of proteins. Phys. Rev. Lett. 2012, 109 (4), 048104 10.1103/PhysRevLett.109.048104. 23006112

[ref32] Foguel D.; Silva J. L. Cold denaturation of a repressor-operator complex: the role of entropy in protein-DNA recognition. Proc. Natl. Acad. Sci. U S A 1994, 91 (17), 8244–8247. 10.1073/pnas.91.17.8244. 8058788 PMC44582

[ref33] Adinolfi S.; Trifuoggi M.; Politou A.; Martin S.; Pastore A. A structural approach to understanding the iron-binding properties of phylogenetically different frataxins. Hum. Mol. Genet. 2002, 11, 1865–1877. 10.1093/hmg/11.16.1865. 12140189

[ref34] Piotto M.; Saudek V.; Sklenár V. Gradient-tailored excitation for single-quantum NMR spectroscopy of aqueous solutions. J. Biomol. NMR 1992, 2, 661–665. 10.1007/BF02192855. 1490109

[ref35] Pons J. L.; Malliavin T. E.; Delsuc M. A. Gifa V. 4: A complete package for NMR data set processing. J. Biomol. NMR 1996, 8, 445–452. 10.1007/BF00228146. 20859778

[ref36] Hawley S. A. Reversible Pressure-Temperature Denaturation of Chymotrypsinogen. Biochemistry 1971, 10, 2436–2441. 10.1021/bi00789a002. 5557794

[ref37] Ravindra R.; Winter R. On the temperature-pressure free-energy landscape of proteins. ChemPhysChem 2003, 4, 359–365. 10.1002/cphc.200390062. 12728550

[ref38] Van Deuren V.; Yang Y.-S.; de Guillen K.; Dubois C.; Royer C. A.; Roumestand C.; Barthe P. Comparative assessment of NMR probes for the experimental description of protein folding pathways with high-pressure NMR. Biology 2021, 10 (7), 656–668. 10.3390/biology10070656. 34356511 PMC8301334

[ref39] Fossat M. J.; Dao T. P.; Jenkins K.; Dellarole M.; Yang Y.; McCallum S. A.; Garcia A. E.; Barrick D.; Roumestand C.; Royer C. A. High-resolution mapping of a repeat protein folding free energy landscape. Biophys. J. 2016, 111, 2368–2376. 10.1016/j.bpj.2016.08.027. 27926838 PMC5153537

[ref40] Dubois C.; Herrada I.; Barthe P.; Roumestand C. Combining high-pressure perturbation with nmr spectroscopy for a structural and dynamical characterization of protein folding pathways. Molecules 2020, 25, 5551 10.3390/molecules25235551. 33256081 PMC7731413

[ref41] Vehlow C.; Stehr H.; Winkelmann M.; Duarte J. M.; Petzold L.; Dinse J.; Lappe M. CMView: Interactive contact map visualization and analysis. Bioinformatics 2011, 27, 1573–1574. 10.1093/bioinformatics/btr163. 21471016

[ref42] Jumper J.; Evans R.; Pritzel A.; Green T.; Figurnov M.; Ronneberger O.; Tunyasuvunakool K.; Bates R.; Žídek A.; Potapenko A.; Bridgland A.; Meyer C.; Kohl S. A. A.; Ballard A. J.; Cowie A.; Romera-Paredes B.; Nikolov S.; Jain R.; Adler J.; Back T.; Petersen S.; Reiman D.; Clancy E.; Zielinski M.; Steinegger M.; Pacholska M.; Berghammer T.; Bodenstein S.; Silver D.; Vinyals O.; Senior A. W.; Kavukcuoglu K.; Kohli P.; Hassabis D. Highly accurate protein structure prediction with AlphaFold. Nature 2021, 596 (7873), 583–589. 10.1038/s41586-021-03819-2. 34265844 PMC8371605

[ref43] Roche J.; Royer C. A.; Roumestand C. exploring protein conformational landscapes using high-pressure NMR. Methods Enzymol. 2019, 614, 293–320. 10.1016/bs.mie.2018.07.006. 30611428

[ref44] Adrover M.; Esposito V.; Martorell G.; Pastore A.; Temussi P. A. Understanding cold denaturation: the case study of Yfh1. J. Am. Chem. Soc. 2010, 132, 16240–16246. 10.1021/ja1070174. 20979399

[ref45] Adrover M.; Martorell G.; Martin S. R.; Urosev D.; Konarev P. V.; Svergun D. I.; Daura X.; Temussi P.; Pastore A. The role of hydration in protein stability: comparison of the cold and heat unfolded states of Yfh1. J. Mol. Biol. 2012, 417, 413–424. 10.1016/j.jmb.2012.02.002. 22342930

[ref46] Wishart D. S.; Bigam C. G.; Holm A.; Hodges R. S.; Sykes B. D. 1H, 13C and 15N random coil NMR chemical shifts of the common amino acids. I. Investigations of nearest-neighbor effects. J. Biomol. NMR 1995, 5, 67–81. 10.1007/BF00227471. 7881273

[ref47] Saotome T.; Doret M.; Kulkarni M.; Yang Y. S.; Barthe P.; Kuroda Y.; Roumestand C. Folding of the Ig-like domain of the Dengue virus envelope protein analyzed by high-hydrostatic-pressure nmr at a residue-level resolution. Biomolecules 2019, 9, 309 10.3390/biom9080309. 31357538 PMC6723665

[ref48] Lahfa M.; Mouhand A.; de Guillen K.; Barthe P.; Kroj T.; Padilla A.; Roumestand C. Does a similar 3d structure mean a similar folding pathway? The presence of a c-terminal α-helical extension in the 3D structure of MAX60 drastically changes the folding pathway described for other MAX-effectors from Magnaporthe oryzae. Molecules 2023, 28, 6068 10.3390/molecules28166068. 37630320 PMC10460046

[ref49] Martin S. R.; Esposito V.; De Los Rios P.; Pastore A.; Temussi P. A. Cold denaturation of yeast frataxin offers the clue to understand the effect of alcohols on protein stability. J. Am. Chem. Soc. 2008, 130 (30), 9963–9970. 10.1021/ja803280e. 18593164

[ref50] Dubois C.; Planelles-Herrero V. J.; Tillatte-Tripodi C.; Delbecq S.; Mammri L.; Sirkia E. M.; Ropars V.; Roumestand C.; Barthe P. Pressure and chemical unfolding of an α-helical bundle protein: The GH2 domain of the protein adaptor GIPC1. Int. J. Mol. Sci. 2021, 22, 3597 10.3390/ijms22073597. 33808390 PMC8037465

[ref51] Harish B.; Gillilan R. E.; Zou J.; Wang J.; Raleigh D. P.; Royer C. A. Protein unfolded states populated at high and ambient pressure are similarly compact. Biophys. J. 2021, 120 (12), 2592–2598. 10.1016/j.bpj.2021.04.031. 33961866 PMC8390852

[ref52] Dellarole M.; Caro J. A.; Roche J.; Fossat M.; Barthe P.; García-Moreno E B.; Royer C. A.; Roumestand C. evolutionarily conserved pattern of interactions in a protein revealed by local thermal expansion properties. J. Am. Chem. Soc. 2015, 137, 9354–9362. 10.1021/jacs.5b04320. 26135981

[ref53] Rouget J. B.; Schroer M. A.; Jeworrek C.; Pühse M.; Saldana J. L.; Bessin Y.; Tolan M.; Barrick D.; Winter R.; Royer C. A. Unique features of the folding landscape of a repeat protein revealed by pressure perturbation. Biophys. J. 2010, 98, 2712–2721. 10.1016/j.bpj.2010.02.044. 20513416 PMC2880709

[ref54] Castro I. H.; Pignataro M. F.; Sewell K. E.; Espeche L. D.; Herrera M. G.; Noguera M. E.; Dain L.; Nadra A. D.; Aran M.; Smal C.; Gallo M.; Santos J. Frataxin structure and function. Subcell. Biochem. 2019, 93, 393–438. 10.1007/978-3-030-28151-9_13. 31939159

[ref55] Puglisi R.; Brylski O.; Alfano C.; Martin S. R.; Pastore A.; Temussi P. A. Quantifying the thermodynamics of protein unfolding using 2D NMR spectroscopy. Commun. Chem. 2020, 3, 100 10.1038/s42004-020-00358-1. 33718626 PMC7116895

[ref56] Puglisi R.; Karunanithy G.; Hansen D. F.; Pastore A.; Temussi P. A. The anatomy of unfolding of Yfh1 is revealed by site-specific fold stability analysis measured by 2D NMR spectroscopy. Commun. Chem. 2021, 4 (1), 127 10.1038/s42004-021-00566-3. 35243007 PMC7612453

[ref57] Berner F.; Kovermann M. Including the ensemble of unstructured conformations in the analysis of protein’s native state by high-pressure nmr spectroscopy. Angew. Chem., Int. Ed. Engl. 2024, 63 (27), e202401343 10.1002/anie.202401343. 38656763

[ref58] Privalov P. L. Stability of proteins. Proteins which do not present a single cooperative system. Adv. Protein Chem. 1982, 35, 1–104. 10.1016/S0065-3233(08)60468-4. 6762066

[ref59] Privalov P. L. Stability of proteins small globular proteins. Adv. Protein Chem. 1979, 33, 167–241. 10.1016/S0065-3233(08)60460-X. 44431

[ref60] Babu C.; Hilser V.; Wand A. Direct access to the cooperative substructure of proteins and the protein ensemble via cold denaturation. Nat. Struct. Mol. Biol. 2004, 11, 352–357. 10.1038/nsmb739. 14990997

[ref61] Whitten S. T.; Kurtz A. J.; Pometun M. S.; Wand A. J.; Hilser V. J. Revealing the nature of the native state ensemble through cold denaturation. Biochemistry 2006, 45 (34), 10163–10174. 10.1021/bi060855+. 16922491

[ref62] Wang S.; Gu J.; Larson S. A.; Whitten S. T.; Hilser V. J. Denatured-state energy landscapes of a protein structural database reveal the energetic determinants of a framework model for folding. J. Mol. Biol. 2008, 381 (5), 1184–1201. 10.1016/j.jmb.2008.06.046. 18616947 PMC6583914

[ref63] Hilser V. J.; García-Moreno E B.; Oas T. G.; Kapp G.; Whitten S. T. A statistical thermodynamic model of the protein ensemble. Chem. Rev. 2006, 106 (5), 1545–1558. 10.1021/cr040423+. 16683744

[ref64] Bitonti A.; Puglisi R.; Meli M.; Martin S. R.; Colombo G.; Temussi P. A.; Pastore A. recipes for inducing cold denaturation in an otherwise stable protein. J. Am. Chem. Soc. 2022, 144, 7198–7207. 10.1021/jacs.1c13355. 35427450 PMC9052743

[ref65] Sanfelice D.; Puglisi R.; Martin S. R.; Di Bari L.; Pastore A.; Temussi P. A. Yeast frataxin is stabilized by low salt concentrations: cold denaturation disentangles ionic strength effects from specific interactions. PLoS One 2014, 9, e95801 10.1371/journal.pone.0095801. 24802807 PMC4011691

