# Supplementary material for: Understanding the Relationship between Pressure and Temperature Unfolding of Proteins
Source: JACS Au. 2025 Mar 20;5(4):1940–55. doi: 10.1021/jacsau.5c00185 (PMC12042054; doi:10.1021/jacsau.5c00185)
Supplement: Supplementary file 1 — au5c00185_si_001.pdf [file au5c00185_si_001.pdf]

Supplementary Materials

Understanding the relationship between pressure and temperature unfolding of proteins

Christian Roumestand[1]*, Erika Dudas[2], Rita Puglisi[3], Antonino Calió[4], Philippe Barthe[1], Piero Andrea Temussi[5], Annalisa Pastore[3]*

[1]Centre de Biologie Structurale, Univ Montpellier, CNRS UMR 5048, INSERM U 1054, 34090 Montpellier, France

[2]European Synchrotron Radiation Facility, Ave des Martyrs, 38000 Grenoble, France

[3]King's College London, 5 Cutcombe Rd, SE59RT London, UK

[4]University Grenoble Alps, 38000 Grenoble, France

[5]Department of Chemistry, Universita' Federico II, 80100 Napoli, Italy

Analysis of the reference structures of Yfh1

For the analysis of the folding pathway according to (Roche et al., 2012), it is necessary to refer to a three-dimensional structure of the protein. Of the seven entries available in PDB for Yfh1, 2ga5 is an NMR structure that has serious geometric distortions caused by misassignment: as previously demonstrated (Vilanova et al., 2014), the authors mistraced the sequential assignment, mixing resonances from the folded and the unfolded states. This caused strain and a poor geometry. The other six structures, solved by crystallography at *ca.* 3 Å resolution, are those of a mutant in which a well conserved tyrosine (Tyr73) was substituted by an alanine (Söderberg et al., 2011). This mutation enhances the tendency of the protein to form large aggregates in the presence of high concentrations of iron but affects the structure of the N-terminal helix. The NMR and crystallographic structures superpose with a 2.5 Å rmsd (on residues 75 to 172 using the numbering of the mature protein and of the 3oeq entry), with maximal differences in the orientation of the C-terminal helix and in the structure of the 6th and 7th putative ß-strands. To clarify the issue, we used AlphaFold (Jumper et al., 2021) to generate a model that could be consistent with the structures of all Yfh1 orthologues. The AlphaFold2.2 software yielded models with a high degree of confidence: only eight out of 123 residues had a pLDDT (predicted local distance difference test) score <0.9, all located at the N-terminus of

the molecule, which has been demonstrated to be intrinsically disordered and flexible (Popovic et al., 2015). This model superposes with the crystal structure with a 1.1 Å rmsd in the globular region (residues 75-172), as compared to a 2.2 Å rmsd with the NMR structure. A better superposition is observed for the ß-sheet between the X-ray structure (2oeq) and the AlphaFold model, but the lengths of the ß-strands are not always the same. The X-ray structure also lacks, due to the structural distortions induced by the Tyr73Ala mutation, the first two helical turns of the N-terminal helix (L70-E76) that are present both in the AlphaFold model and in the NMR structure. The crystal structure lacks the two last residues (S172, Q173), yielding to a shortening of the C-terminal helix where an additional turn is observed both in the NMR structure and on the AlphaFold model. For these reasons, we considered that the AlphaFold model captures all the details of the NMR structure but with a better geometry. We thus considered this model our reference structure,-although, for comparison, we also used the NMR structure, finding comparable results (data not shown).

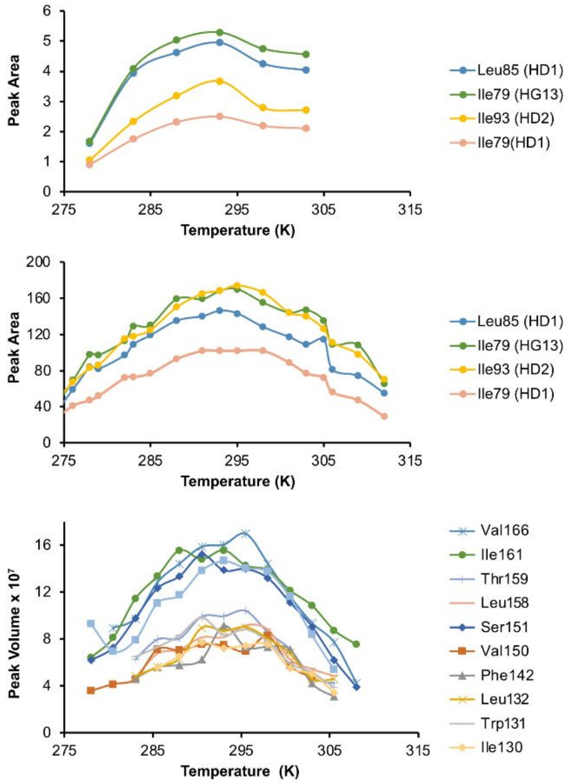

Figure S1 – Dependence of the areas or volumes of resonances at room pressure as a function of temperature. Top: Plot of the peak areas of the ring-current shifted resonances of the 1D spectrum as a function of temperature. Ring-current shifted resonances are particularly diagnostic of the protein core because they correspond to residues, typically methyl protons but not only, that are shifted as compared to their average position in a random coil protein because persistently sitting spatially close to aromatic groups (Perkins and Wuethrich, 1979). We had previously used these resonances to get the stability curves (i.e. the plot of ΔG versus temperature) at 1 bar, from which the thermodynamic parameters of the unfolding process can be obtained (Pastore et al., 2007; Martin et al., 2008; Puglisi et al., 2021). The resonances were assigned according to the BMRB entry 19991. **Middle**: The same but from Pastore et al. (2007). **Bottom**: Peak volumes from 2D HSQC for the amide protons of specific residues that were identified as buried and thus as being diagnostic of the unfolding process (Puglisi et al., 2019). The data were obviously collected on different protein preparations, produced by different researchers. The excellent agreement of the profiles gives us a measure of the reproducibility of the process.

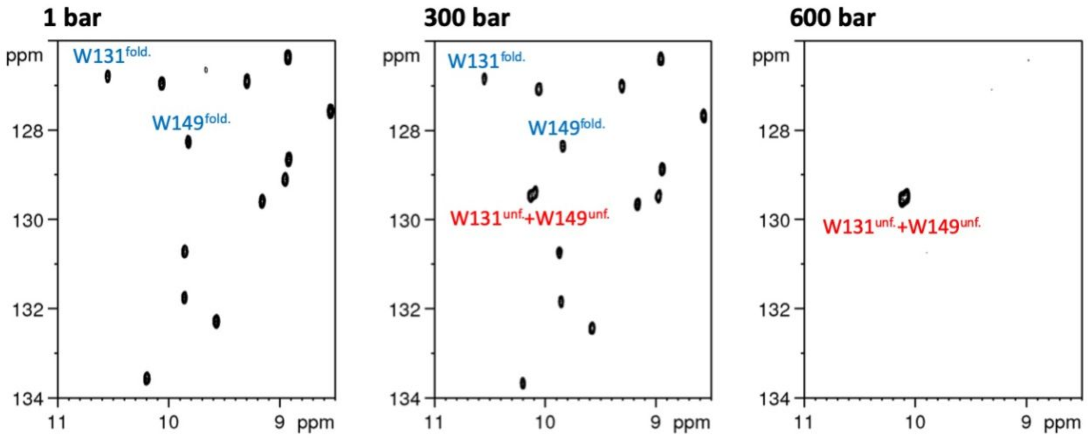

Figure S2. Evolution of the indole resonances of the two tryptophan residues of Yfh1 as a function of pressure (temperature: 20°C). Folded resonances are labelled in blue, unfolded ones in red.

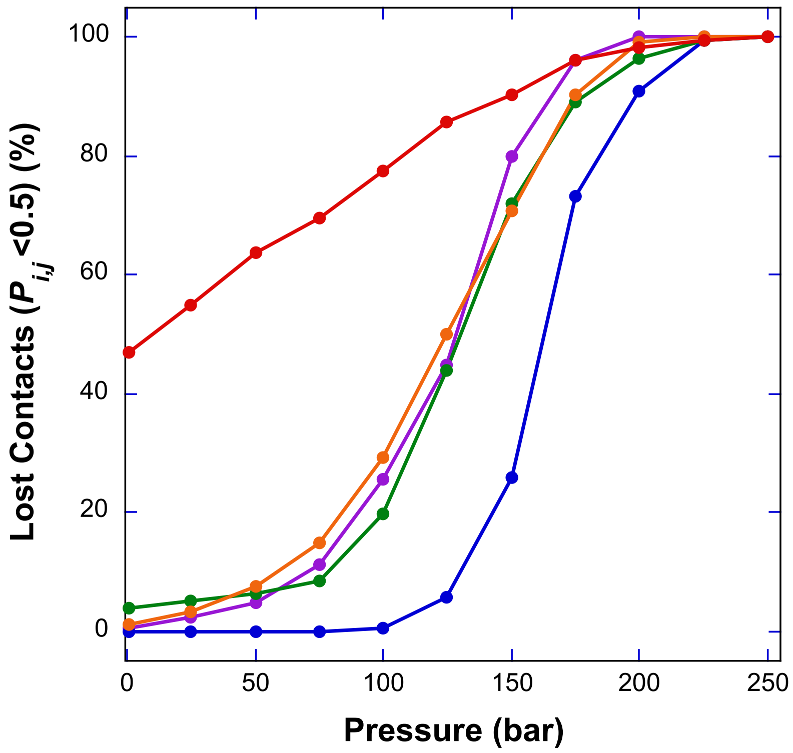

Figure S3. Evolution of the Loss of Contacts as a function of pressure during the unfolding transition of Yfh1. The plot shows the data collected at 283 K (violet curve), 288 K (blue curve), 293 K (green curve), 298 K (orange curve) and 303 K (red curve). The considered contacts involve residues for which residue-specific pressure denaturation curves can be obtained at the five temperatures. A contact between two residues *i* and *j* was considered as lost when the corresponding calculated probability of contact $P_{i,j}$ was found to be >0.5.

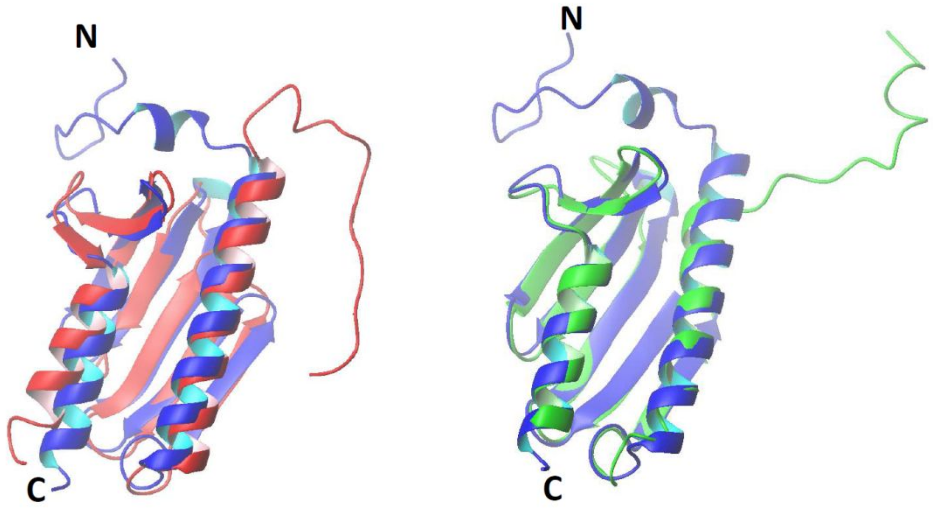

Figure S4. Comparison between the experimental structures and the AlphaFold model of Yfh1. Superimposition of the 3D structures (ribbon representations) of the AlphaFold model of Yfh1 (blue ribbon) with the corresponding NMR solution structure (2ga5, left) and the X-ray structure (3eoq) (green ribbons, right).

References

Perkins, S.J., Wüthrich, K. (1979) Ring current effects in the conformation dependent NMR chemical shifts of aliphatic protons in the basic pancreatic trypsin inhibitor. (1979) Biochimica et Biophysica Acta Protein Structure, 576, 409-423,

Roche J, Caro, JA, Norberto DR, Barthe P, Roumestand C, Schlessman JL, Garcia AE, Royer CA. (2012) Cavities determine the pressure unfolding of proteins. Proc. Natl. Acad. Sci. USA 109, 6945–6950.
